# A probability model for estimating age in young individuals relative to key legal thresholds: 15, 18 or 21-year

**DOI:** 10.1007/s00414-024-03324-x

**Published:** 2024-09-18

**Authors:** Nina Heldring, Ali-Reza Rezaie, André Larsson, Rebecca Gahn, Brita Zilg, Simon Camilleri, Antoine Saade, Philipp Wesp, Elias Palm, Ola Kvist

**Affiliations:** 1 Department of Forensic Medicine, Swedish National Board of Forensic Medicine, Retzius Väg 5, 171 65 Stockholm, Sweden; 2 Department of Oncology-Pathology, Karolinska Institutet, Retzius V. 3, 171 77 Stockholm, Sweden; 3 Paindrainer, Medicon Village, 223 81 Lund, Sweden; 4 Faculty of Dentistry, Oral and Craniofacial Sciences, Tower Wing, Guys’ Hospital St Thomas Street, London, England; 5 Department of Orthodontics, Faculty of Dental Medicine, Lebanese University, Beirut, Lebanon; 6 Department of Radiology, LMU University Hospital, LMU Munich, Marchioninistraße 15, 81377 Munich, Germany; 7 Munich Center for Machine Learning (MCML), Geschwister‑Scholl‑Platz 1, 80539 Munich, Germany; 8 Pediatric Radiology Department, Karolinska University Hospital, Stockholm, Sweden; 9 Department of Women’s and Children’s Health, Karolinska Institute, Stockholm, Sweden

**Keywords:** Age distribution, Bayesian theorem, Biological variation, Population, Forensic anthropology, Validation study

## Abstract

Age estimations are relevant for pre-trial detention, sentencing in criminal cases and as part of the evaluation in asylum processes to protect the rights and privileges of minors. No current method can determine an exact chronological age due to individual variations in biological development. This study seeks to develop a validated statistical model for estimating an age relative to key legal thresholds (15, 18, and 21 years) based on a skeletal (CT-clavicle, radiography-hand/wrist or MR-knee) and tooth (radiography-third molar) developmental stages. The whole model is based on 34 scientific studies, divided into examinations of the hand/wrist (15 studies), clavicle (5 studies), distal femur (4 studies), and third molars (10 studies). In total, data from approximately 27,000 individuals have been incorporated and the model has subsequently been validated with data from 5,000 individuals. The core framework of the model is built upon transition analysis and is further developed by a combination of a type of parametric bootstrapping and Bayesian theory. Validation of the model includes testing the models on independent datasets of individuals with known ages and shows a high precision with separate populations aligning closely with the model’s predictions. The practical use of the complex statistical model requires a user-friendly tool to provide probabilities together with the margin of error. The assessment based on the model forms the medical component for the overall evaluation of an individual’s age.

**Supplementary Information:**

The online version contains supplementary material available at 10.1007/s00414-024-03324-x.

## Introduction

There are many shortcomings in all medical age assessments that are being applied in different countries. No current method can determine an exact chronological age (CA) due to the individual variations in biological development. Still, there are practical needs to assess age in various legal contexts with minimal error rates. Age estimation is relevant for pre-trial detention and sentencing in criminal cases as well as part of the evaluation in asylum processes to protect the rights and privileges of minors. The European Asylum Support Office (EASO) recommends using the least intrusive examination for medical age assessments methods in their practical guide [1] with radiation free procedures argued to be preferable in children and young adults. The lack of validated or standardized methods has rendered countries within or outside the EU to choose various methods of medical age assessment [1, 2]. In addition, the mission differs slightly between countries in terms of the questions that are expected to be answered as well as which party carries out the task. In many nations, adopting a minimum age concept is a prevalent strategy aimed at minimizing the risk of misclassifying minors. However, this strategy overlooks the potential drawbacks of erroneously classifying adults as minors. Such consequences include misallocation of resources intended for minors to adults and hindrance to the proper administration of justice, as adults may escape prosecution in criminal cases. Probability methods provide a most likely age distribution based on a large reference population rather than an indeterminable CA. The overall approach to provide a probability of an individual being below or above a certain age includes, as a first step, to examine the developmental stages of a selected skeletal component together with the wisdom tooth, and then comparing this to the age distribution of the reference population of the same sex and developmental stages. The probabilities are supplemented with the margin of error, represented by the minor portion of the reference population distribution in relation to the chosen age threshold. The order of magnitude of the margin of error reflects the certainty level of the assessment. Notably, there is a knowledge gap of how one can objectively use multiple anatomical locations and statistical models to estimate the age of an individual more accurately. Having validated models ensures fairness and accuracy as far as possible in legal proceedings. This study seeks to develop and present a validated statistical model for estimating an age relative to key legal thresholds (15, 18, and 21 years) based on skeleton (CT-clavicle, radiography-hand/wrist, MR-knee) and teeth radiography-third molar) developmental stages.

## Methods

### Data included in the model

A literature search was conducted to identify scientific studies investigating hand/wrist, third molar, distal femur or clavicle maturity in relation to age. After removal of duplicate articles and categorization based on title and abstract, full text articles were read and the following exclusion criteria were applied:

1) Imaging method other than radiography (hand/wrist, third molar), MRI (distal femur), CT (clavicle). 2) Incomplete data: the study does not present all the data needed to recreate individual-based data. 3) Different staging than Greulich & Pyle (hand/wrist), Demirjian (third molar) Krämer (Distal femur), Schmeling (Clavicle). 4) The study population does not include ages on both sides of the 15- and 18-year boundaries (Distal femur only). 5) Other anatomical structure than selected indicators. 6) Previously published results, e.g. analysis or review of previous data. 7) Post-mortem study population. 8) Full text not available in English, Swedish, Danish or Norwegian. 9) Study based on data that is not available. 10) Study population includes individuals with a disease that may affect skeletal maturity. 11) Study population has uneven age distribution according to Chi-square test (type 3 data only).

All the hand/wrist studies investigated skeletal age based on radiographs where the developmental stages are classified according to Greulich & Pyle [3]. Studies were identified through targeted searches on PubMed using the strategy (skeletal matur* OR ossifi* OR age estimat* OR forensic age OR age asses* OR age determin*) AND (radiography OR radiograph* OR x-ray OR ionizing) AND (Greulich OR Pyle) and Embase, which generated 727 studies. The data included in the model were obtained from 15 hand/wrist studies that met the criteria (Table 1).

**Table 1:** Studies included in the probability model

Indicator	Study	Men	Women	Country	Age span (men)	Age span (women)	Method	Type of data
Hand	Alcina 2018 [52]	590	560	Spain	0–19	0–18	x-ray	Type 1b
Hand	Bala 2010 [53]	80	80	India	8–14	8–14	x-ray	Type 4
Hand	Büken 2007 [54]	251	241	Turkey	11–20	11–19	x-ray	Type 4
Hand	Cantekin 2012 [55]	342	425	Turkey	7–18	7–18	x-ray	Type 1b
Hand*^~^	Chaumoitre 2017 [56]	1423	1191	France	1–21	1–20	x-ray	Type 3
Hand	Dembetembe 2012 [57]	131	-	South Africa	13–22	13–22	x-ray	Type 1b
Hand	Elamin 2017 [58]	487	627	Sudan	1–28	2–37	x-ray	Type 4
Hand	Hackman 2013 [59]	249	157	Scotland	1–20	1–20	x-ray	Type 1b
Hand	Koc 2001 [60]	225	-	Turkey	7–17	-	x-ray	Type 4
Hand	Mora 2001 [61]	265	269	USA	0–19	0–19	x-ray	Type 4
Hand	Paxton 2013 [62]	276	130	Australia	1–18	1–18	x-ray	Type 1b
Hand	Soudack 2012 [63]	375	304	Israel	1–18	1–18	x-ray	Type 1b
Hand*	Tisé 2011 [64]	359	125	Italy	11–19	11–19	x-ray	Type 3
Hand	van Rijn 2001 [65]	278	294	Netherlands	5–20	5–20	x-ray	Type 1b
Hand	Zabet 2015 [66]	100	90	France	10–18	10–19	x-ray	Type 1b
Distal femur*	Ekizoglu 2020 [67]	335	314	Turkey	12–30	12–30	MRI	Type 3
Distal femur	Krämer 2014 [68]	166	124	Germany	10–31	10–31	MRI	Type 3
Distal femur	Ottow 2017 [69]	326	335	Germany	12–25	12–26	MRI	Type 3
Distal femur	Saint-Martin 2014 [70]	214	-	France	14–20	-	MRI	Type 2
Clavicle*	Ekizoglu 2015 [71]	362	141	Turkey	10–35	10–35	CT	Type 3
Clavicle	Franklin 2015 [72]	185	148	Australia	10–35	10–35	CT	Type 2
Clavicle*	Pattamapaspong 2015 [32]	249	160	Thailand	11–29	11–29	CT	Type 3
Clavicle	Uysal 2017 [73]	399	202	Turkey	10–35	10–35	CT	Type 2
Clavicle	Zhang 2015 [74]	370	382	China	15–25	15–25	CT	Type 3
Third molar	Duangto 2017 [75]	877	990	Thailand	8–23	8–23	x-ray	Type 2
Third molar	Hassan 2021 [76]	170	180	Egypt	14–24	14–24	x-ray	Type 3
Third molar	Hegde 2016 [77]	664	475	India	5–16	5–16	x-ray	Type 2
Third molar	Johan 2012 [78]	540	540	Malaysia	14–25	14–25	x-ray	Type 2
Third molar	Kasper 2009 [79]	804	1019	USA (Latino)	12–22	12–22	x-ray	Type 2
Third molar	Lee 2009 [80]	786	964	South Korea	7–22	7–24	x-ray	Type 2
Third molar	Li 2012 [81]	989	1089	China	5–23	5–23	x-ray	Type 2
Third molar	Liu 2018 [82]	1012	1196	China	8–23	8–23	x-ray	Type 2
Third molar	Lopez 2013 [83]	236	315	Brazil	15–23	15–23	x-ray	Type 3
Third molar	Quispe 2017 [84]	102	106	Peru	14–22	14–22	x-ray	Type 3

Studies included in the probability model. MRI = Magnetic resonance imaging, x-ray = Radiological X-ray, CT = Computed tomography. * Only data for women were used as the age distribution was not evenly distributed for men. ~ Some stages were excluded as these did not fulfil the normal distribution assumption according to the research article

All the dental studies related the development of the third molar in the lower jaw, imaged with plain radiographs and classified by Demirjian, to CA in the study populations. Dental studies were identified from the summaries previously made in BioAlder 1.3 [4–7]. A total of 58 articles were identified, all of which were read in full text and 10 studies met the criteria and were included in the model (Table 1).

The distal femur studies related the development of the upper knee joint (distal femur), examined by magnetic resonance imaging (MRI) with field strength of at least 1.5T and T1 weighting, to CA after classification according to Krämer 2014 [8]. Studies were identified from Heldring et al. 2022 [7], supplemented with articles from an internal literature monitoring procedure on distal femur studies. A total of 27 studies were identified and read in full text and 4 of these met the criteria and were selected for inclusion (Table 1.)

Original clavicle studies where the development of clavicles according to Schmeling’s staging (1–5) [9] and CA was studied, were identified. This was done by a literature search in PubMed using the string ((skeletal matur* OR ossifi* OR age estimat* OR forensic age OR age asses* OR age determin*) AND (clavicle OR medial epiphysis OR medial end OR medial clavicular epiphysis OR sternal epiphysis OR sternal end) AND (CT scan OR computed tomography OR CT OR scanner OR Schmeling’s method OR “chest radiographs” OR “forensic radiology”) which generated 296 articles and 5 clavicle studies met the criteria for inclusion (Table 1). 

### Data extraction and simulating population age distributions

The method of data extraction is adapted to how the data is presented in each study. In order to fit the probabilistic model to the datasets, all data must include a list with known CA and corresponding developmental stage for each individual. The format of type 1 data provides CA presented together with the development stage for each individual either in a table by the authors (type 1a) or extracted from a figure with PlotDigitizer (type 1b) [10], hence can be included without recreation.

However, datasets where both CA and corresponding developmental stage are not reported for each individual require recreation of individual-based datasets. Type 2 data are reported as the frequency of different stages within integer age intervals, either as counts or as fractions together with the total number of individuals for the different intervals. Individual-based data is recreated by calculating the number of individuals with a specific stage in each of the age-cohorts and CAs are assigned randomly within each age interval assuming a uniform distribution. If minimum and maximum of CA for a given developmental stage is provided in addition to the frequency data, the simulated uniform values are further limited to this specified interval.

Type 3 data present the number of individuals at each stage, alongside essential statistical measures such as the min, max and lower, median and upper quartile of the CA within each stage (type 3b), or the mean and standard deviation for each stage (type 3a). In the case of type 3a data, a normal distribution is used to generate the individual ages, however, if an age range [*a, b*] is additionally specified for each specific stage by the study, a truncated normal distribution is fitted to the reported values. The *truncorm* package (version 1.0–9) in R was used [11] to perform this. For type 3b data, which reports the quantiles of the measured age distributions for each stage, a normal distribution of CA is assumed, for every stage *s*. A truncated normal is fitted through a numerical optimization process that minimizes the errors between the quantiles of the simulated truncated normal distribution and quantiles reported in the study. In the full dataset, CA from type 3a and 3b datasets are therefore simulated with either a normal or truncated normal distribution using the estimated parameters as described above. Further details on this approach and the truncated normal can be found in Supplementary Appendix.

Type 4 data reports mean age, standard deviation, and Pearson’s correlation for an age-cohort of both the CA and skeletal age. To simulate populations, the process includes a two-step approach, as described in Bleka et al. [5]. In short, the additional information provided by the Pearson’s correlation coefficient is incorporated by fitting a multivariate normal distribution to the data, including the conditional dependence between CA and stage. The resulting bivariate normal distribution is then used to recreate the CA and the stages for each individual in the study. All resulting statistics in this report are derived from 10,000 simulated populations, unless stated otherwise.

### The probability model

The first step in generating the probabilities is to obtain an estimate of chronological stage *s* through finding the probability of stage given age, *P(S* = *s | A*), by fitting ordinal/logistic regression models to the datasets of each individual developmental indicator. In the second step, these results are used in equation 1,

1 $$P\left(A\:\right|S=s)=\frac{P\left(S=s\right|A) \, P(A)}{\int\nolimits_b^a{}\;P\left(S=s\right|y) \, P\left(y\right)dy}$$

to obtain the inverse probability of age given stage, *P(A | S* = *s)* for each indicator*.* As this equation only depends on *P(S* = *s | A),* assuming a uniform prior, we can find the normalizing factor in the denominator by requiring the total area of the probability density function (PDF) to be one. Finally, we end up with a probability density function *P(A | S* = *s)* for each stage/combination of stages *s,* which can be integrated to find the relevant statistics, such as the probability of stage *s* for being below or above a certain age threshold. This two-step approach also using re-created population data was taken to minimize the influence of age mimicry [12]. The probability of being below 15, 18 or 21-year thresholds is calculated based on all 10,000 simulations with bootstrapping for each stage, and the 50th percentile is selected as the estimate. From the bootstrap sample, we also determine a 95% confidence interval for the calculated statistics based on the 2.5th and 97.5th percentile. In addition, the probability of the one-year age-cohorts within the assumed age distribution is computed by applying the 50th percentile value from all simulated 10,000 populations.

### Prior age distribution

The selected uniform prior ensures that all information is derived from the data in the posterior distribution as the purpose is to generate the conditional PDF without any subjective influence. This approach with a non-informative prior requires a defined lower and upper limit of the uniform distribution being determined by the assumed age range within the model. Based on the endpoint of the second-to-last stage for hand/wrist, 20 years of age for females and 21 for males was chosen as the upper bound (Roberts et al. (2015) [13]. In order to avoid an increased risk of type 1 errors (identifying children as adults) in the third molar model, the upper limit is set in accordance with Knell et al. (2009) [14] and Olze et al. (2010) [15], at the age when 50% of the population reaches stage H (21 years for both genders) due to the wide distribution of the second-to-last stage G. The lower bound for both the hand/wrist as well as the third molar model is set to 7 years for both sexes. Data from clavicle studies typically span ages 10–35, and it is noted that stage 4 of the clavicle can still be detected among 35-year-olds for both genders. Similar to the third molar model, the upper limit for the clavicle model is set at the age when 50% of the population reaches the last development stage (stage 5). Hence, the assumed age range was considered between 10–30 years for females and 10–32 years for men, for the clavicle model. For distal femur, we adopted an age range of 15–21, as proposed in Heldring et al. (2022) [7].

### Additional assumptions when combining two indicators

In order to obtain an estimate of CA when the stages of several different developmental indicators are combined, we assume that stages are conditionally independent from each other. Previous probability models similar to this one assume a conditional independence between skeleton development and third molar development [5, 7, 16] based on studies investigating hand/wrist and third molar development [17–19]. The study that is comparing models that included or excluded a co-dependence between indicators on a combination dataset concluded that there was no statistically significant improvement in the accuracy of age estimation when including a conditional dependence between indicators [5]. However, this assumption does not apply between skeletal indicators, rendering the calculation of probabilities in those combinations inaccurate.

The probability of one skeleton indicator being in stage *s*_*s*_ and the third molar indicator being in stage *s*_*t*_ for a given age, can be expressed as.

2 $$P\left(S_s=s_s,S_t=s_t\right|A)=P\left(S_s=s_s\right|A)\cdot P\left(S_t=s_t\left|A\right.\right)$$

assuming conditional independence between the indicators. To obtain the reverse conditional probability, probability of age given stage *s* (Eq. 2) is applied analogous to the calculations in Eq. 1.

For the combined clavicle and third molar model, the upper limit is set to 26.0 years, as the data is truncated at this age for the third molar model. The upper limit is set to 21 years for both females and males for the third molar and hand/wrist combination, as well as the third molar and distal femur model. In addition, the dichotomous distal femur model in combination with third molar is based on the age range 15–21 years and includes the relevant Demirjian stages D-H.

### Model selection

Two candidate ordinal regression models, cumulative and continuous-ratio (CR), with either logit or probit for the linking functions and using either parallel or non-parallel odds-ratios were considered (Supplementary Appendix). This is similar to models previously described in the BioAlder tool [5].

The best model was selected based on a goodness-of-fit of the data for each indicator and gender combination. For each 10,000 populations, the Akaike information criterion (AIC) [20] was computed for every model combination and the final model was selected based on the lowest median AIC value. The choice of AIC was motivated by its ability to penalize the addition of extra parameters estimated in the ordinal model, thereby balancing model complexity. This process was carried out individually for each indicator and gender, yielding a total of 8 distinct models. Both the cumulative and the CR model will be equivalent to a simple logistic regression model for indicators with only two separate stages as in the distal femur model.

The model was written in R (Version 4.3.1) [21]. The ordinal/logistic regression models were fitted by applying the *vglm* function in the VGAM (Version 1.1–9) package [22]. The different conditional PDFs were created by extracting the corresponding parameters from the ordinal/logistic models followed by applying Bayes’ theorem. To calculate the area under the curve of the conditional PDF for a given threshold or one-year cohorts, the *integrate* function was applied. The method for estimating the prediction intervals (PI) of the CA is described in the Supplementary Appendix.

### Collection of validation populations

The access to independent datasets is mainly dependent on other researchers. In our initial search for studies to be included when building the model, we identified studies where data is presented in a format that was not suitable or had a high risk of age mimicry. We invited some of the authors of these studies and additional studies found in later searches to share their primary data (CA, development stage and gender) to be used as independent validation populations (Table 2). In addition, an independent study of clavicles with CT was performed. The study was retrospective in its design with all cases extracted from Karolinska University Hospital, Stockholm, and approved by the Swedish Ethical Review Authority (Dnr 2024–00531-01). Individuals aged 17.0 to 25.0 years examined during routine clinical practice and with known CA and sex were selected. Scans with poor image quality and individuals with an injury or a skeletal disease that could affect clavicle development were excluded. Selected scans were subsequently assessed with regard to development stage in agreement with the Schmeling staging system [9, 23] on the most developed side by one radiologist with 14 years of musculoskeletal (MSK) radiology experience and 8 years with focus on pediatric MSK radiology experience.

**Table 2:** List of validation datasets

Indicator	Study	Men	Women	Country	Age span (men)	Age span (women)	Method	Type of data
Hand	Maggio 2018 [85]	104	108	Australia	0–25	0–25	x-ray	Type 1a
Hand	Saade 2017 [28]	115	108	Lebanon	8–16	8–15	x-ray	Type 1a
Hand	Zafar 2010 [86]	167	84	Pakistan	0–18	0–18	x-ray	Type 1a
Distal femur*	Socialstyrelsen 2018 [87]	217	178	Sweden	14–22	14–22	MRI	Type 1a
Third molar	Jayaraman 2022 [88]	189	186	USA (Latino)	8–17	7–17	x-ray	Type 1a
Third molar	Knell 2009 [14]	551	622	Switzerland	15–22	15–22	x-ray	Type 1a
Third molar	Malta collection	553	651	Malta	8–26	7–25	x-ray	Type 1a
Third molar	Saade 2017 [28]	1113	119	Lebanon	8–16	8–15	x-ray	Type 1a
Clavicle	Swedish collection	199	201	Sweden	17–24	17–25	CT	Type 1a
Clavicle	Wesp 2024 [50]	28	22	Germany	15–29	15–29	CT	Type 1a

List of validation datasets including number of individuals, age span and country. *Observations with MRI T1 TSE sequence (bridging study)

### Validation of the statistical model with independent datasets

We used the true development stages of the independent individual observations for the classification of whether they fall below or above the 15-, 18- or 21-year age threshold limits. This classification process involves selecting a cutoff point of the given probability where probabilities below the cutoff will classify the individual as above the threshold while probabilities above the cutoff will generate a classification of the individual as below the age threshold. While a common method involves ROC curve analysis to determine an optimal cutoff point to maximize sensitivity and specificity, the chosen cutoff point of 0.35 was based on being an acceptable error of the mean for a final evaluation. This strategy consequently leads to minimizing type 1 errors (classifying underage as overage) and as a consequence will classify more individuals being over the age threshold as under than the opposite if applied. The individuals and proportions being correctly or incorrectly classified are visualized and presented in distribution-plots, point-plots, bar graphs and line-graphs (Fig. 3, 4, 5, and 6 and Supplementary Fig. 12). The distribution of the collected validation populations is visualized as interpolated kernel density estimator (KDE) of the different study distributions and all the studies combined (Supplementary Fig. 1 (a-b)). The KDE is fitted with the geom_density function in the ggplot2 package [24].

In order to calculate the minimum sample size required to estimate the precision of the models, the *pmsampsize* function from the pmsampsize package [25] was used in R. To calculate the minimal sample size needed for external validation of prediction models with a binary outcome (correct or incorrect classification) [26, 27] included a conservative outlook with a c-statistics of 0.85 and a prevalence of 0.15, meaning 15% misclassification of events are expected. This resulted in 195 individuals for a validation sample size for males and females, respectively.

## Results

### Data included in the model

Observations from approximately 27,000 individuals from 6 geographic regions are included in the model (Table 1 and Supplementary Table 1).

### Selected model

We found that the continuation-ratio model with logit link function and a non-parallel slope coefficient provided the best fit for the clavicle and third molar model (both sexes). A continuation-ratio model with probit link function and a non-parallel slope coefficient fitted the data best for the hand/wrist model in both sexes. For distal femur, where only two stages are used (not closed/closed), logistic regression with a logit link function for both sexes was the best fit and used in the final model. A graphic representation of how the fitted parametric regression model relates to the calculated semi-annually proportion of underlying data (non-parametric), calculated as the fraction of individuals with a specific stage in the simulated datasets, is presented in Supplementary Fig. 2–8.

We refrained from log-transforming the CA variable to avoid potentially increasing complexity within the model, as the non-parallel fit gives the posterior distributions more flexibility as they were being estimated and because of the assumption of normal distributions among stages. This is in contrast to previous models where a parallel slope coefficient for all models and log-transformation was applied [16]. We demonstrate that certain third molar stages, fitted with the KDE from one of the randomly generated populations compared with its fitted PDF, appear to be approximately normal distributed (Supplementary Fig. 1 (c-n)) when the influence of age mimicry is low, i.e. where the chronological age of the data is approximately uniformly distributed (Supplementary Fig. 1 (a-b)). 

### Age prediction model

**Fig. 1:**
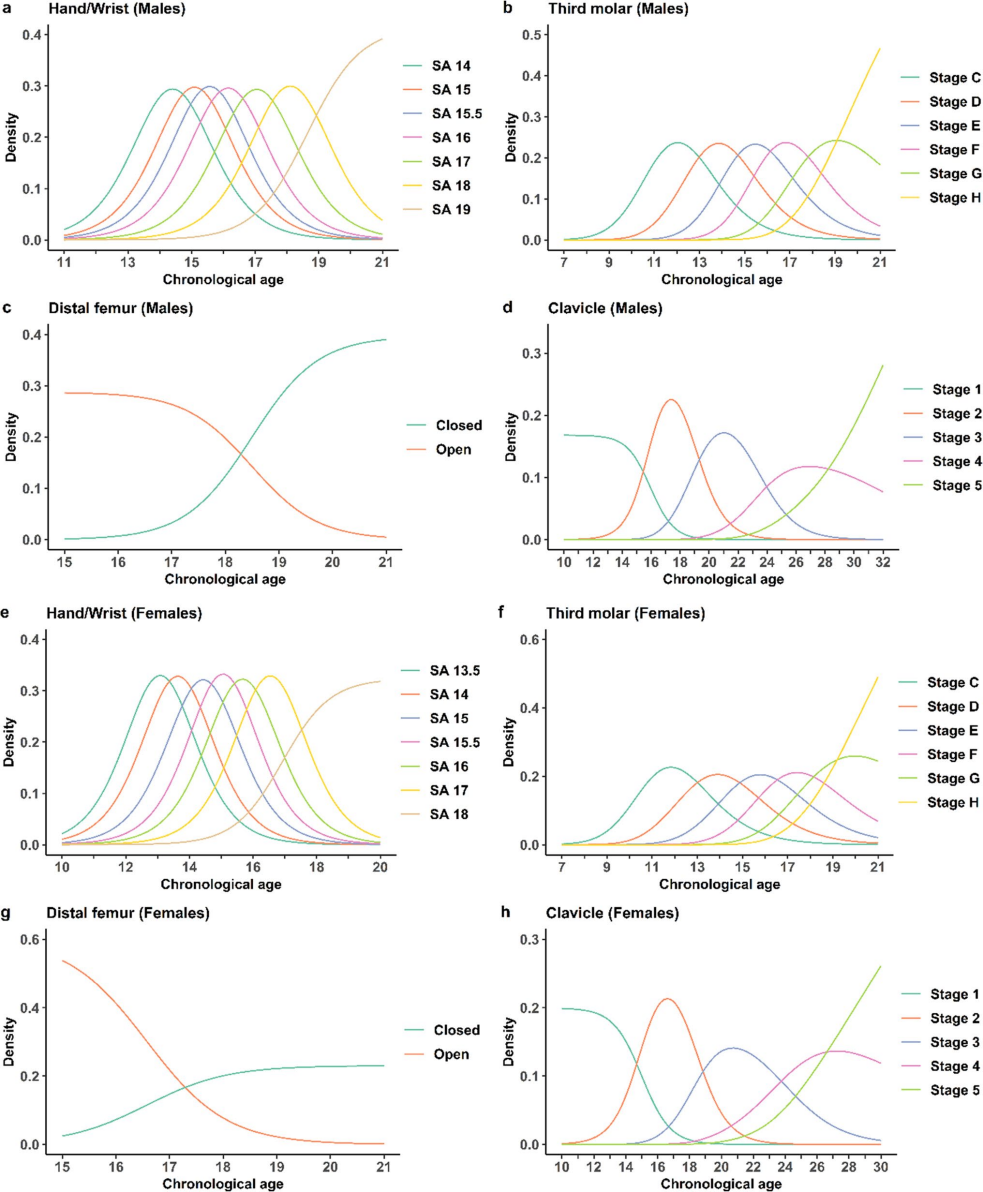
Probability density functions. Age distributions for hand/wrist, third molar, distal femur and clavicle stages for male (**a**-**d**) and female (**e**–**h**) individuals in terms of density of developmental stage hand/wrist skeletal age 14–19 and 13.5–18 respectively (Greulich & Pyle) (**a** and **e**), third molar stage C-H (Demirjian) (**b** and **f**), distal femur reached final stage or not (Krämer) (**c** and **g**) and clavicle stage 1–5 (Schmeling) (**d** and **h**)

The estimated 75% and 95% PI’s of CA for the hand/wrist and third molar stages of development are shown in Supplementary Fig. 9, separately (a) and in combination (b), as the median from 10,000 simulated populations. The age distributions are wider when using a single indicator compared to combining the third molar with hand/wrist, indicating that multifactorial age estimations are more accurate compared to using a single anatomical site. This is also seen for the combination with the distal femur (Supplementary Fig. 10) or clavicle (Supplementary Fig. 11). The PDF’s for hand/wrist, third molar, distal femur, and clavicle assuming normally distributed ages for each indicator and stage are shown for males (a-d) and females (e–h) in Fig. 1. The distributions display one randomly selected distribution from the 10,000 generated populations for each stage.

### Combining indicators

**Fig. 2:**
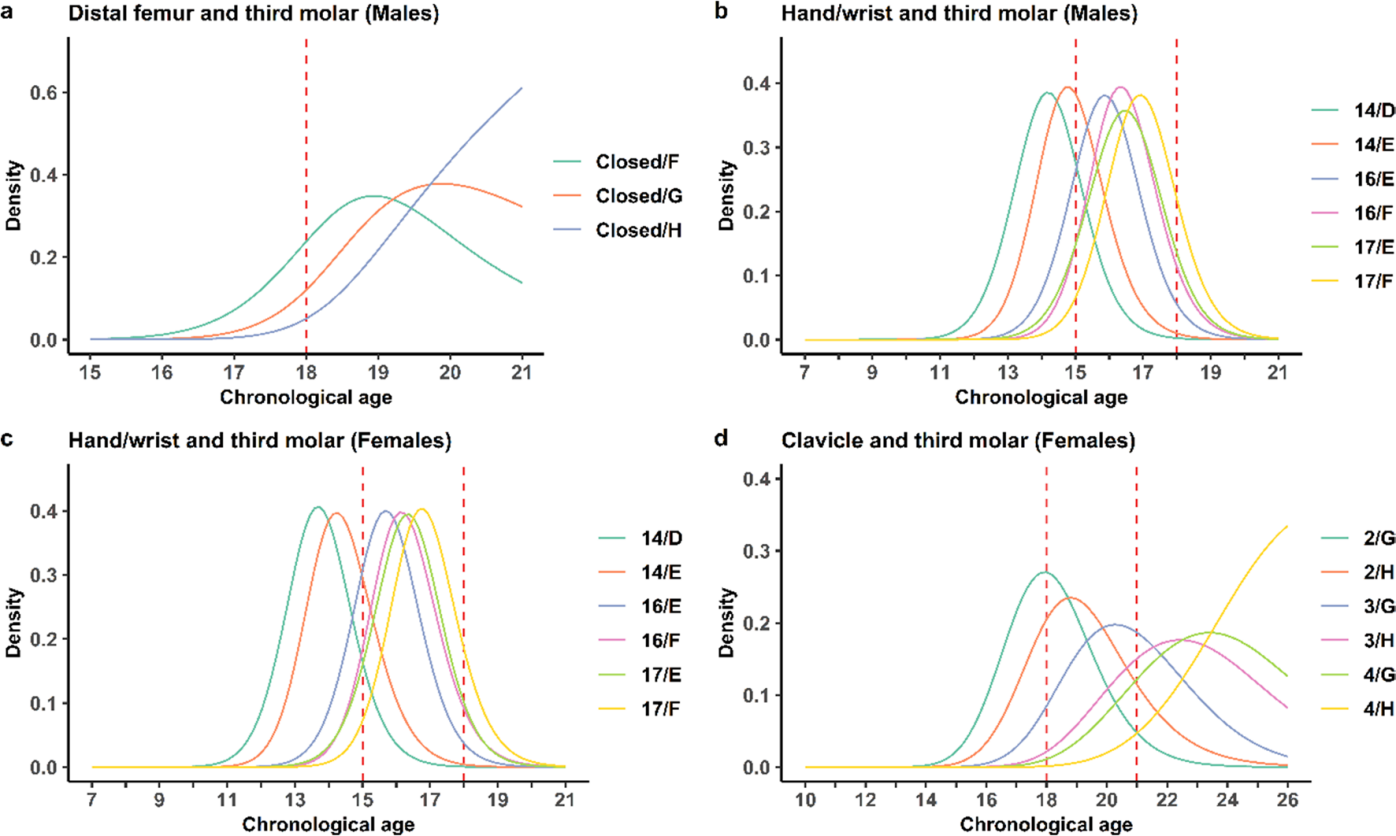
Probability density functions for combinations. Age distributions for selected combinations in terms of density of developmental stages for distal femur in combination with third molar (males) (**a**), hand/wrist in combination with third molar (males) (**b**), hand/wrist in combination with third molar (females) (**c**), and clavicle in combination with third molar (females) (**d**). Red dotted line represents age thresholds of interest

From the known probability of being in a stage given age, we derived the conditional PDF for age within this stage by using Bayes’ theorem (Eq. 1). The assumption of conditional independence does not apply between skeletal indicators, rendering the three skeletal indicators inappropriate to combine. Hence, the current combinations are third molar with either one of the skeletal indicators. Age distributions for selected combinations are shown in Fig. 2 for males (a and b) and females (c and d). The probability of age in relation to a certain threshold is represented by the part of a specific combination’s distribution being on either side of the age limit. The distribution as well as probabilities are affected by the chosen upper age limit for each indicator. A sensitivity analysis was performed with several upper age limits (Table 3, hand/wrist and third molar, Supplementary Table 2 clavicle and Supplementary Table 3 clavicle and third molar). We observe that the probabilities of being under 18 years of age is only minimally affected if the upper age limit is increased for the combination of hand/wrist and third molar (Table 3). We also noted that the probabilities of being under the 21-year threshold for stage 4 or 5 in the clavicle model do not vary significantly when changing the upper boundary between 30 and 35 years (Supplementary Table 2). This demonstrates that the chosen distribution predicts reliable probabilities.

**Table 3:**
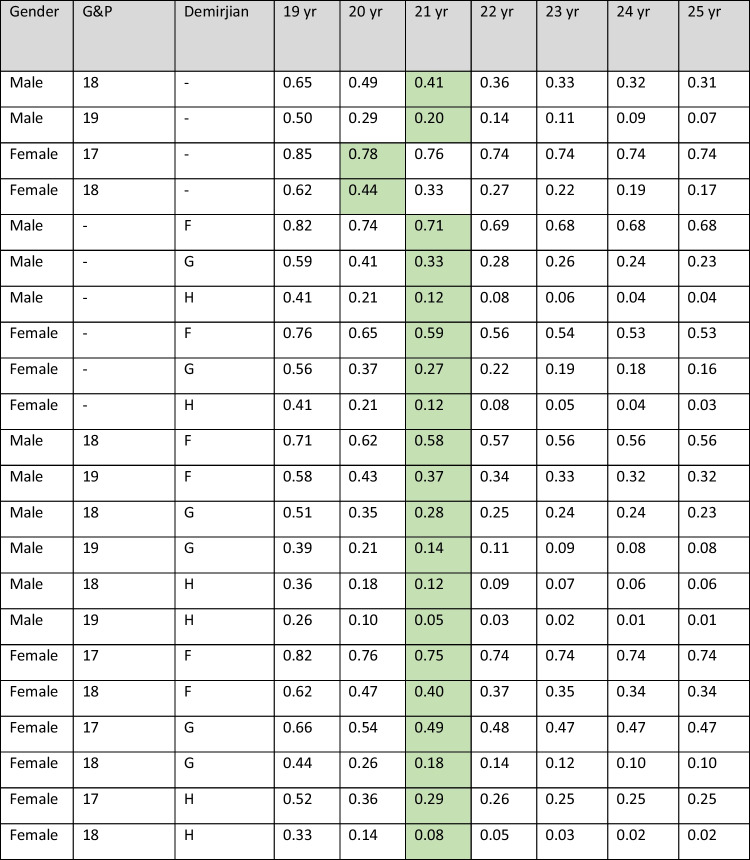
Sensitivity analysis of upper age limits for hand/wrist and third molar stages

Sensitivity analysis of upper age limits. Probabilities of being under 18 when the upper age limit is varied on the uniform distribution for hand/wrist or third molar stages and for the combination of these indicators. The green color indicates the upper limit applied in the statistical model

### Validation with independent test populations

To assess how well the model performs on independent data, a number of datasets for populations of known age have been collected and used for validation (Table 2). Aside from the Swedish collection of a clavicle dataset that was collected specifically for the purpose of the validation of the model, the datasets are from published studies or collections, kindly provided by authors and researchers upon contact. Each indicator was validated separately, except the combination of third molar and hand/wrist where examination and developmental stage were studied in the same individual for one of the datasets [28].

### Validation of the third molar model

**Fig. 3:**
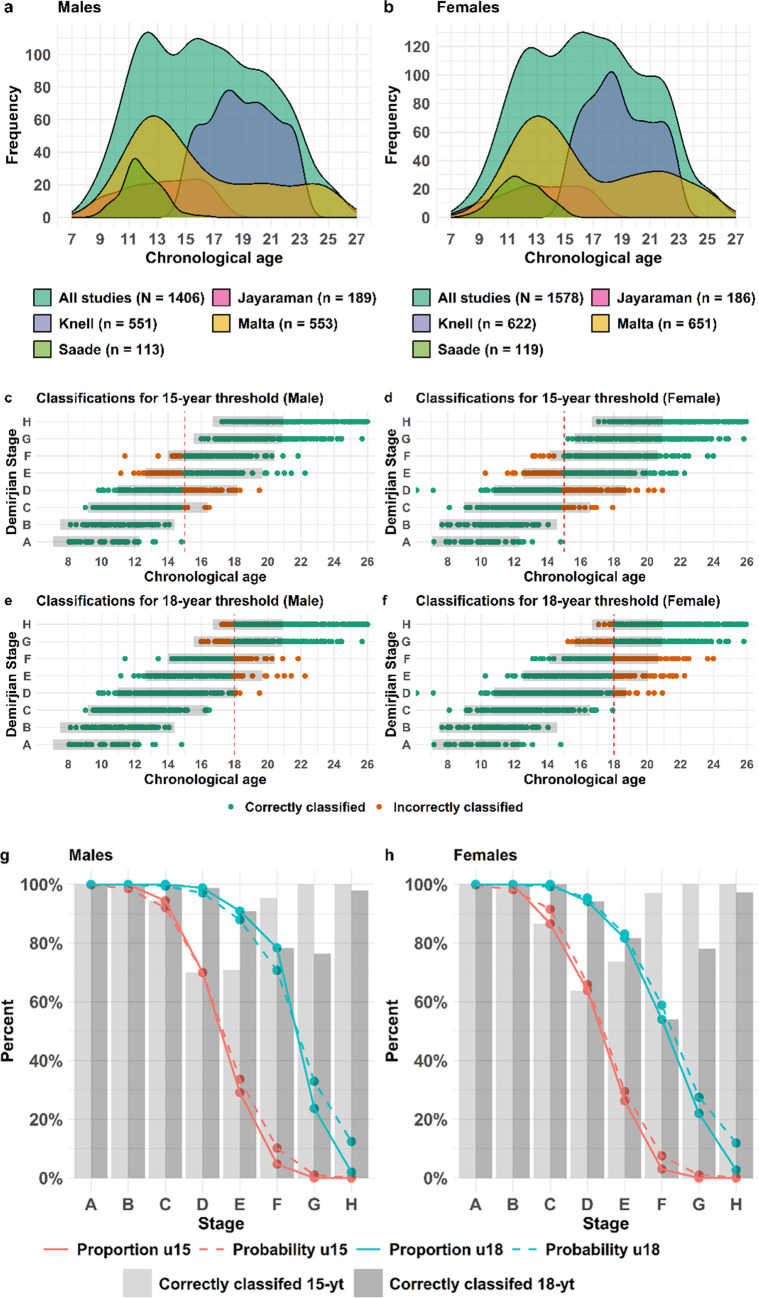
Validation of the third molar model Validation of the third molar model. Distribution of the full validation dataset and the separate studies are shown for males (**a**) and females (**b**). Point plots displaying the chronological age and corresponding Demirjian development stage of the third molar together with classification with regard to the 15-or 18- year threshold for males ((**c**) and (**e**)) and females ((**d**) and (**f**)). Grey bars in (**c-f**) represents the 95% PI for each development stage. The proportion in the validation set (full line) being under 15(orange) or 18 (blue) for each development stage together with the predicted probability according to the statistical model (dashed lines) for males (**g**) and females (**h**). The proportion of the validation set being correctly classified (**g-h**) with regard to the 15-year threshold (light grey bar) and the 18-year threshold (dark grey bar) is displayed for each development stage for males (**g**) and females (**h**)

The validation set for third molar included in total 1406 males (Fig. 3(a)) and 1578 females (Fig. 3(b)), spanning an age interval between 7–26 years (Table 2) and originates from 4 separate datasets (Fig. 3). In total, 93% of the male and 87% of the female populations were correctly classified regarding the 18-year threshold, corresponding to the separate model’s total accuracy (Table 4 and Fig. 3 (c-f)). In addition, the model accuracy with regard to the 15-year threshold is 90% for males and 87% for females (Table 4 (a)). The sensitivity (adults identified as adults) of the male third molar model is 90% and specificity (children identified as children) is 95% for the 18-year threshold, while the positive predictive value (identified as adults that are adults) is 91% and the negative predictive value (identified as children that are children) is 94% (Table 4 (a)). The corresponding sensitivity in the female third molar model is 75% and the specificity 94% (Table 4 (a)). Not surprisingly, very early stages cause few errors in the assessments of both the 15-and the 18-year threshold (Fig. 3 (c-f)). Most of the incorrectly classified individuals are in the development stages C-F for the 15-year threshold and D-H for the 18-year threshold in both males (c and e) and females (d and f). These individuals are fewer compared to correctly classified individuals (Fig. 3 (g-h)), and represent both individuals with an age close to the limit and individuals with either early or late third molar development (Fig. 3 (c-f)). The proportion of the independent population being under 15 (orange full line) or 18 (blue full line) years overlaps almost completely with the predicted probabilities (dashed lines) for the model (Fig. 3 (g-h)), for both males (g) and females (h). This demonstrates a high reliability of the probability model.

**Table 4:** Quantitative reliability of the models

(a)				
Measure	Third molar model			
	Males		Females	
	15-year threshold	18-year threshold	15-year threshold	18-year threshold
Sensitivity	0.90	0.90	0.86	0.75
Specificity	0.89	0.95	0.89	0.94
PPV	0.93	0.91	0.93	0.89
NPV	0.85	0.94	0.78	0.86
Accuracy	0.90	0.93	0.87	0.87
(b)				
Measure	Hand/wrist model			
	Males		Females	
	15-year threshold	18-year threshold	15-year threshold	18-year threshold
Sensitivity	0.81	0.33	0.89	0.00
Specificity	0.92	0.99	0.91	1.00
PPV	0.81	0.67	0.73	-
NPV	0.91	0.96	0.97	0.94
Accuracy	0.88	0.95	0.91	0.94
(c)				
Measure	Hand/wrist model			
	Males		Females	
	15-year threshold	18-year threshold	15-year threshold	18-year threshold
Sensitivity	0.81	0.33	0.89	0.00
Specificity	0.92	0.99	0.91	1.00
PPV	0.81	0.67	0.73	-
NPV	0.91	0.96	0.97	0.94
Accuracy	0.88	0.95	0.91	0.94
(d)				
Measure	Distal femur model			
	Males		Females	
	15-year threshold	18-year threshold	15-year threshold	18-year threshold
Sensitivity	-	0.82	-	0.91
Specificity	-	0.96	-	0.78
PPV	-	0.96	-	0.77
NPV	-	0.82	-	0.92
Accuracy	-	0.88	-	0.84
(e)				
Measure	Clavicle model			
	Males		Females	
	18-year threshold	21-year threshold	18-year threshold	21-year threshold
Sensitivity	0.76	0.59	0.86	0.64
Specificity	0.82	0.96	0.82	0.95
PPV	0.97	0.95	0.97	0.94
NPV	0.32	0.65	0.45	0.69
Accuracy	0.77	0.75	0.85	0.78

Quantitative measures of the model’s reliability for the third molar model (a) hand/wrist model (b) distal femur model (c) clavicle model (d) and the combination of hand/wrist with third molar (d). Quantifications based on the independent dataset validations. Sensitivity (CA above age limit and identified as above the age limit), specificity (CA under age limit identified as under the age limit), positive predictive value ((PPV) individuals identified as above the age limit with CA above the age limit), negative predictive value ((NPV) individuals identified as below the age limit with CA below the age limit) and total accuracy presented for the 15-year and/or 18-year and/or 21-year threshold for the male and female models

### Validation of the hand/wrist model

**Fig. 4:**
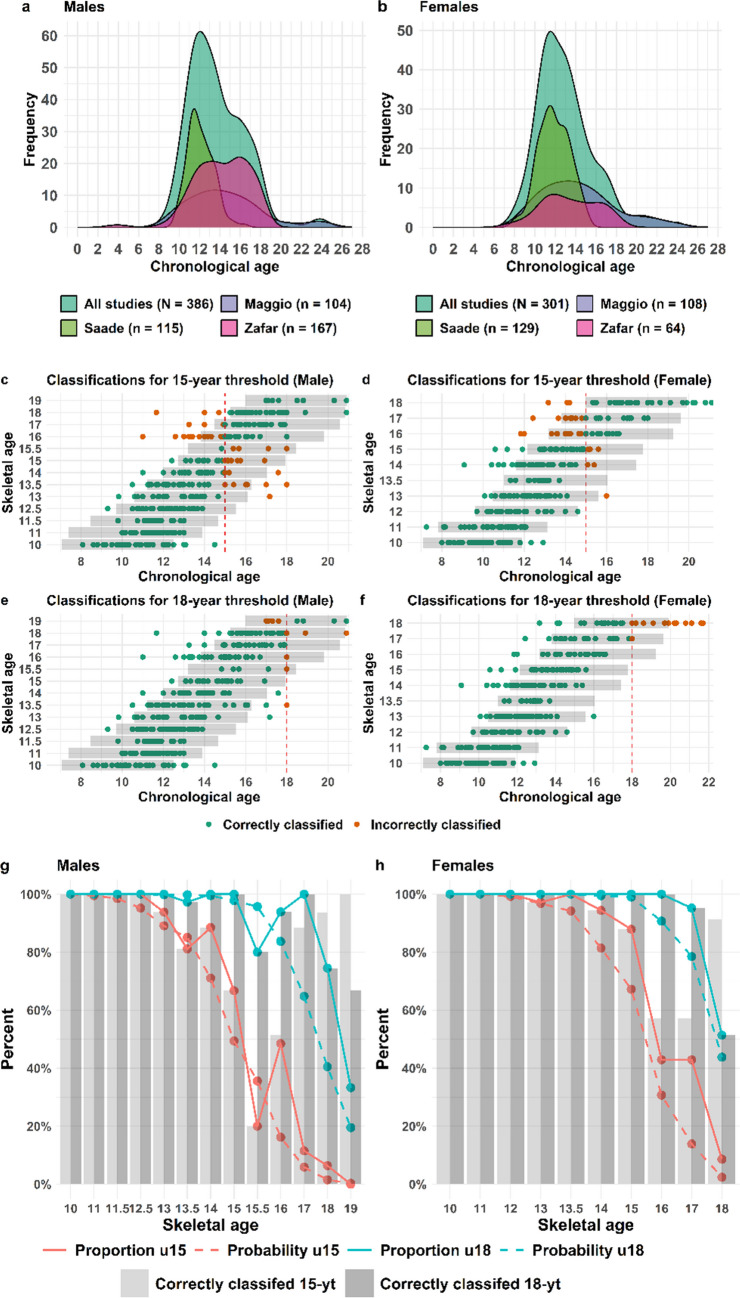
Validation of the hand/wrist model. Distribution of the full validation dataset and the separate studies for males(**a**) and females(**b**). Point plots displaying the chronological age and corresponding G&P development stage of hand/wrist together with classification with regard to the 15- or 18- year threshold for males (**c**) and (**e**) and females (**d**) and (**f**). Grey bars in (**c**-**f**) represents the 95% PI for each development stage in the model. The proportion in the validation set (full line) being under 15 (orange) or 18 (blue) for each development stage together with the predicted probability according to the statistical model (dashed lines) for males (**g**) and females (**h**). The proportion of the validation set being correctly classified with regard to the 15-year threshold (light grey bar) and the 18-year threshold (dark grey bar) displayed for each development stage in the model for males (**g**) and females (**h**)

In total, 386 males (Fig. 4 (a)) and 301 females (Fig. 4 (b)), spanning an age interval between 7–26 years and originating from 3 separate datasets (Fig. 4 (a-b)) are included in the independent validation set for hand/wrist. What distinguishes the hand/wrist model from the dental model is that it is suitable for assessing the 15-year threshold but is of limited use for the 18-year threshold as the last developmental stage begins before the age of 18 to a large extent (Fig. 1 (a, e)). In total, 88% of the male and 91% of the female populations were correctly classified regarding the 15-year threshold (Table 4 (b)). Similar to the third molar model, incorrectly classified individuals are not found in the early development stages but have reached skeletal age (SA) 13 up to 18 (Fig. 4 c-f) in both males (c) and females (d). The incorrectly classified individuals are fewer compared to correctly classified (Fig. 4) in both males (g) and females (h) except for SA 16 and 17 in females with regard to the 15-year threshold where it is equal (h). With regard to the 18-year threshold, the model has an acceptable precision when it comes to below 18 (Fig. 4(e–f)), while the development stages of hand/wrist do not seem to allow for accurate age estimations with regard to above18 years of age. The proportion of individuals being under 15 (orange full line) or 18 (blue full line) in the independent validation population of the hand/wrist model basically follows the probabilities of being under 15 (orange dashed line) or 18 (blue dashed line) according to the model for males and females (Fig. 4 (g-h). However, the non-smoothness of the curves reflects the limited number of individuals being in some of the SA development stages in the validation population. The sensitivity (aged over 15 identified as aged over 15) of the male hand/wrist model is 81% and specificity (under 15 identified as under 15) is 92% for the 15-year threshold (Table 4 (b)). The corresponding sensitivity of the female hand/wrist model is 89% and specificity is 91% for the 15-year threshold (Table 4 (b)). Keeping in mind that the proportion of individuals above 18-years of age in the independent population is limited (Fig. 4 (c-f)), the total accuracy with regard to the 18-year threshold for the male model is 93% and for the female model, 90% (Table 4 (b)).

### Validation of the distal femur model

**Fig. 5:**
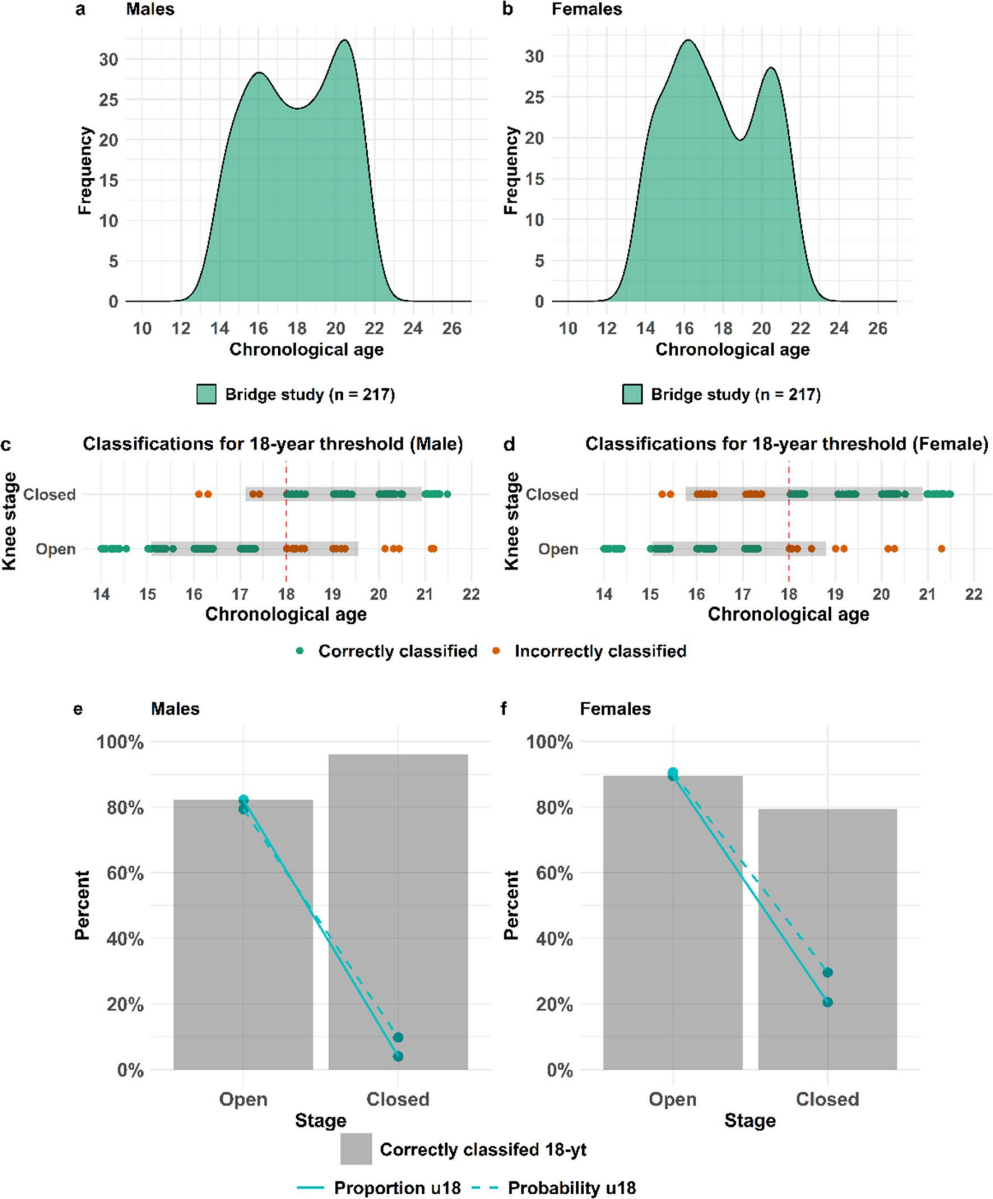
Validation of the distal femur model. Distribution of the full validation dataset for males (**a**) and females(**b**). Point plots displaying the chronological age and corresponding dichotomous development stage of the distal femur together with classification with regard to the 18-year threshold for males (**c**) and females (**d**). Grey bars in (**c-f**) represents the 95% PI for each development stage in the model. The proportion in the validation set (full line) being under 18 (blue) for the development stages together with the predicted probability according to the statistical model (dashed lines) for males (**e**) and females (**f**). The proportion of the validation set being correctly classified with regard to the 18-year threshold (dark grey bar) displayed for the two development stages for males (**e**) and females (**f**)

The validation set of the distal femur model included a population of total 217 males (Fig. 5 (a)) and 217 females (Fig. 5 (b)), spanning an age interval between 12–23 years and originates from one dataset (Fig. 5 (a-b) and Table 2). The distal femur model is based on dichotomous development where the Krämer stages 1–3 are defined as open and 4–5 are defined as closed [7, 8], rendering the model useful exclusively for the 18-year threshold. In total 88% of the independent male and 84% of the female population were correctly classified with regard to the 18-year threshold (Table 4 (c)) corresponding to the accuracy. The incorrectly classified individuals are in minority compared to correctly classified (Fig. 5) in both males (e) and females (f). In regard to the 18-year threshold, the model has an acceptable precision when it comes to men (Fig. 5 (c) and (e)), while a closed distal femur in women generates a lower precision (Fig. 5 (d) and (f)). The proportion of individuals being under 18-years of age (blue full line) in the independent population used for validation of the distal femur model basically follows the probabilities of being under 18-years of age (blue dashed line) according to the model (Fig. 5) for males (e) and females (f). The sensitivity (adults identified as adults) of the male distal femur model is 82% and specificity (children identified as children) 96% for the 18-year threshold (Table 4 (c)). The corresponding sensitivity in the female third molar model is 89% and specificity 80% (Table 4 (c)).

### Validation of the clavicle model

**Fig. 6:**
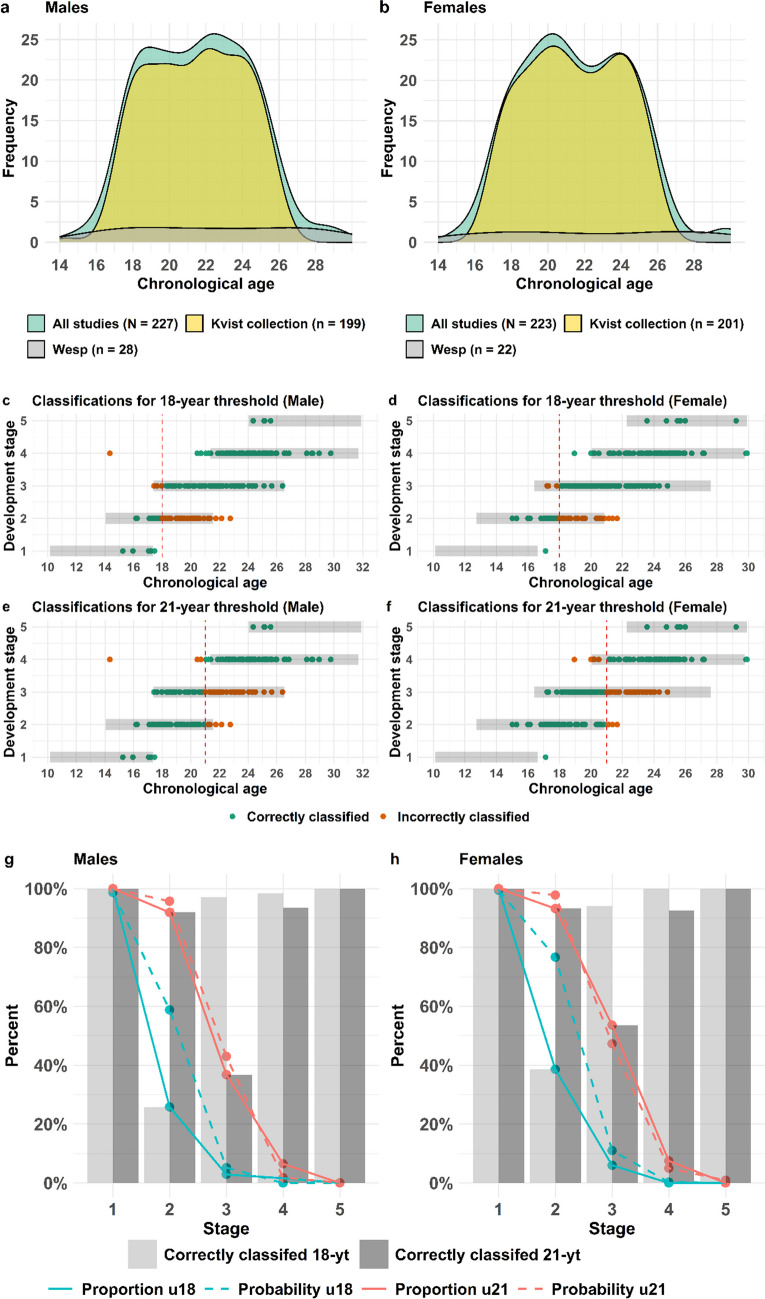
Validation of the clavicle model. Distribution of the full validation dataset for males (**a**) and females(**b**). Point plots displaying the chronological age and corresponding dichotomous development stage of the clavicle together with classification with regard to the 18- and 21- year threshold for males (**c**) and females (**d**). Grey bars in (**c-f**) represents the 95% PI for each development stage in the model. The proportion in the validation set (full line) being under 18 (blue) or 21 (orange) for the development stages together with the predicted probability according to the statistical model (dashed lines) for males (**e**) and females (**f**). The proportion of the validation set being correctly classified with regard to the 18-(grey bar) or 21-year threshold (dark grey bar) displayed for the five development stages for males (**e**) and females (**f**)

The validation set of the clavicle model included a population of total 227 males (Fig. 6 (a)) and 223 females (Fig. 6 (b)), spanning an age interval between 14–30 years and originates from two datasets (Fig. 6 (a-b) and Table 2). Being a skeletal indicator that still develops after 18-years of age renders the clavicle model particularly useful for the 21-year threshold. The validation has been performed for both the 18- and the 21-year threshold. In total 77% of the male and 85% of the female validation population were correctly classified with regard to the 18-year threshold and 75% of the males and 78% of the females to the 21-year threshold (Table 4 (d)) corresponding to the accuracy. The sensitivity (above 21 identified as above) of the male clavicle model is 59% and the specificity (below 21 identified as below 21) is 96% for the 21-year threshold (Table 4 (d)). The corresponding sensitivity in the female clavicle model is 64% and specificity 95% (Table 4 (d)). The incorrectly classified individuals, with regard to the 21-year threshold is mainly individuals in development stage 3 (Fig. 6) for both males (e and g) and females (f and h). For the 18-year threshold, the incorrectly classified individuals are mainly in development stage 2. The proportion of individuals being under 21-years of age (orange full line) in the independent population used for validation of the clavicle model basically follows the probabilities of being under 21-years of age (orange dashed line) according to the model (Fig. 6) for males (g) and females (h), indicating a high reliability of the prediction model. In regard to the 18-year threshold, the validation (blue full line) deviates more from the probabilities according to the prediction model (dashed blue lines) indicating a lower precision compared to the 21-year threshold (orange) (Fig. 6 (g and h).

### Validating the model on a test set with both third molar and hand/wrist

The precision of the age estimation increases when the result from multiple developmental indicators are combined, which corresponds to how the model is recommended to be used in practice. This means that the result from the independent models underestimates the real precision when used in practice. Here, we test our model against one dataset where both third molars and hand/wrist development has been examined in the same individuals, along with CA. The validation data included an independent population of total 106 males and 116 females (Supplementary Fig. 12 (a-b) and Table 2, spanning an age interval between 8–16 years (Supplementary Fig. 12). Classification with Demirjian’s method of the lower left third molar together with the Greulich &Pyle grading of the hand skeleton were applied on individuals in this Lebanese population [28]. The validation of the combined model is limited in that the validation population mostly includes individuals younger than 15 years. However, it is a valuable dataset in that it confirms the higher specificity as demonstrated by a tighter PI compared to single indicators (Supplementary Fig. 9) and a high number of correctly classified under 15 represented by a high specificity for both males (Supplementary Fig. 12 (c) and Table 4 (e)) and females (Supplementary Fig. 12 (d) and Table 4 (e)). In total 96% of the independent male and 97% of the female populations were correctly classified with regard to the 15-year threshold representing the accuracy (Supplementary Fig. 12 (c-d) and Table 4 (e)).

## Discussion

Reliable methods for age estimation in living individuals are of major importance in legal contexts when birth records or other official identification documents are missing. The main aim of this study is to generate and present a validated statistical model for estimating age in living individuals relative to the 15, 18 or 21-year old thresholds. To our knowledge, this is the first model to include several skeletal indicators combined with third molar development to provide assessments for several age thresholds that has been validated with independent datasets. It could be argued that our model addresses the knowledge gap concerning the objective utilization of multiple anatomical locations and statistical models to enhance the accuracy of estimating an individual’s age. The spectrum of methods recommended by the Study Group on Forensic Age Diagnostics in Münster include radiography examination of the hand/wrist and third molars as well as CT clavicle, which may also be supplemented with MRI of distal femur in the future [29]. However, their recommended approach is to add CT clavicle if hand/wrist is fully developed and to use these examinations in a minimal age concept rather than a probability approach. Their recommended methods also include a physical examination and recording of sexual maturity [29], even though the latter is noticed to be against the EASO recommended guidelines [1]. In the statistical model investigated here, radiography of third molar is combined with either radiography hand/wrist, CT clavicle or MRI distal femur depending on the age threshold of interest. The estimation of age from dental radiographs is one of the most studied and widely used approaches, and the Demirjian staging technique is the most widely used staging method in studies focusing on age estimation [6, 30]. Demirjian’s staging of the wisdom tooth is well suited to assess both the 15- and 18-year threshold (Fig. 1 (b and f). Due to a chosen upper age limit at 21 years for the third molar model, it is not suited to assess the 21-year threshold as a single indicator. However, in combination with the clavicle, a slightly older assumed age distribution has been included in the model that renders it suitable (Fig. 2). The higher age as a chosen upper age limit of the third molar in this combination is motivated by the fact that the PI in the combined model is tighter than the clavicle model alone (Supplementary Fig. 11). Radiography of the hand/wrist is internationally the most widely applied method to assess skeletal development [5, 16, 31]. The development stages of hand/wrist are suitable for assessing the 15-year threshold in males and females and possibly the 18-year threshold in males, based on the development stage distributions (Fig. 1 (a and e)). The dichotomous distal femur model is suitable for the 18-year threshold in males while an open development stage can be used in women to indicate minority status (Fig. 1 (c and g)). The medial clavicle epiphysis is considered useful for the 21-year threshold due to a continued development until around age 30 [32–35] (Fig. 1(d and h)).

To create reliable and detailed assessment models, a much larger data set than typically found in a single study is required. The underlying reference population needs to cover all relevant age cohorts that also allow a Bayesian approach to minimize the effect of age mimicry from the underlying studies [12]. Several probability methods have previously been presented in the literature [5, 7, 16]. All these methods have the advantage of relying on larger reference populations when providing age distributions, unlike other assessment approaches that compare with only one limited study population [36]. None of the models will provide a definite age for an individual but in the case of the probability methods, either an age span [5] or a probability of an age in relation to a threshold [7] will be provided, together with an error rate. These probabilities are the base to form the medical component for the overall assessment of an individual’s age.

It has been argued that population-specific reference data is needed in age assessments. According to current scientific understanding, the ethnicity or genetic-geographic origin of an individual may not significantly impact the dental- or skeletal maturity [37–41]. It is noted that a study by Olze et al. [42] as well as a review on dental age estimation [43] cautions against possible differences in dental aging between populations and ethnicities. However, as pointed out before [7] and shown in Rolseth et al. [6], studies might be subject to age mimicry, meaning that the observed difference between populations is likely to reflect differences in the underlying age distributions of the study population rather than inherent differences in development.

Factors such as stress or living standard have been suggested to influence skeletal development [38, 44, 45]. Consequently, individuals from lower socioeconomic backgrounds undergoing medical age assessments may face the risk of being estimated as younger than their CA. In line with the approach of the BioAlder tool [5], we have opted to incorporate a broad spectrum of individuals from chosen studies into the reference population. This decision aims to encompass the widest possible range of biological variations in age-dependent development, striving for thorough coverage. The single studies covering a single geographic region, socio-economic or other possible influencing factors are argued too small to provide reliable reference populations on their own. The total number of individuals included in the model is high (27,000), but is unequally distributed between the included indicators. The number of studies (34) is limited by covering 6 geographic regions and the main limitation factor is the availability of studies focusing on age in relation to development and fulfilling the pre-set criteria. Similar to the previous statistical models [5, 7], the results in this model are dependent on the assumptions for the underlying age distributions, conditional independence and simulations as well as study selection.

Given the inevitable diversity in underlying studies and limited ethnic representation, a key concern that arises when developing a prediction tool is: how accurately does the tool perform for the individuals we intend to predict? The availability of independent complete data sets is scarce, yet essential to perform a validation of the model compared to real world data. The validation of this model with collected independent populations indicates a high accuracy and precision for all indicators, particularly for the third molar model and the distal femur.

When combining dental and skeletal indicators, only a few individuals were wrongly classified with regard to the 15-year threshold in the validation of the combined third molar and hand/wrist model. Considering that the age span in this validation set is limited to a population almost exclusively under 15-years of age, it is possible to establish an adequate level of precision for these individuals, but not for individuals over 15. It has been concluded that a multifactorial age estimation is more accurate than one based on a single anatomical site [46, 47]. Multifactorial age estimation is also recommended by the Münster-based AGFAD study group [29]. An important consideration of multifactorial age estimation is the risk of increased ionizing radiation to a young individual which is against the EASO guidelines and ALARA (as low as reasonably achievable) principle. However, the availability of datasets containing concurrent grading of third molars with a skeletal indicator in the same individuals is limited, and efforts to simultaneously measure multiple developmental indicators would allow for more robust estimations of model accuracy.

The validation with the independent populations has pinpointed and confirmed the predicted development stages that are associated with the highest uncertainties. For instance, 30–40% of the individuals in third molar development stage D in both males and females are wrongly classified with regard to the 15-year threshold (Fig. 3 (g-h)), and this uncertainty agrees with the prediction provided by the model, that these individuals are below 15, with a margin of error of 30% and 35% for males and females, respectively. When applying the model on individuals with an unknown age, the degree of certainty in the statement needs to reflect the estimated age distribution and the probability of being below or above the age limit together with this margin of error that corresponds to the proportion of the reference population on the other side of the limit. The presented validation allows reliable assessments together with margin of errors to be provided.

To facilitate medical age assessments in routine practice using this complex statistical model, a user-friendly tool is advisable. Such a dashboard has been developed to streamline these assessments by forensic pathologists in Sweden. Dropdown menus allow the assessor to populate the model with the current combination of examinations performed together with gender and development stages. The corresponding distribution of the reference population is then displayed together with 95% PI, probability for the three age thresholds together with probabilities in one-year cohorts. This tool provides the probabilities and the measure of margin of error.

A promising tool for faster and more accurate radiological age assessments are artificial intelligence (AI) approaches [30, 35, 48–50]. Methods using AI necessitate a substantial volume of data for construction and are not exempt from the conventional questions inherent in age assessments, such as biologic variation, the socioeconomic dimension or other factors influencing development. An AI tool, based on third molar development in a Brazilian population, presents a binary assessment with high accuracy of being above or below a specific age threshold [49]. In addition, a high accuracy performing AI-model of age classification with regard to 18, 20, 21 and 22-year thresholds based on clavicle development was recently presented in a Chinese study [35]. Notably, a common feature of these methods is that they achieve a high level of accuracy. Even though additional studies are required, deep learning approaches remain a promising vision for the future following validation on a broader scale.

## Limitations

The complex relationship between skeletal or dental development and CA presents an unavoidable barrier to achieving perfect accuracy in age assessment methods [6, 51]. Even though our approach has been to include a broad spectrum of studies performed in different countries and geographic regions in the reference population, the ethnic and socio-economic variation is still limited. The retrospective nature of data collection and the fact that studies are conducted with slightly different protocols and/or data reporting, may introduce variations. The evaluation of the accuracy and precision of the probability model is limited by the access to independent validation populations where multiple indicators have been measured. Although one of the models is based on magnetic resonance imaging, this tool is not entirely devoid of potentially harmful ionizing radiation.

## Conclusion

In summary, our study presents a validated statistical model for estimating an age relative to key legal thresholds (15, 18, and 21 years) based on a skeleton (CT-clavicle, radiography-hand/wrist or MR-knee) and teeth (radiography-third molar) developmental stages allowing to provide reliable assessments with margin of errors. This probability model provides a most likely age distribution based on a large reference population rather than an indeterminable CA. The assessment based on the model generated probabilities form the medical component for the overall assessment of an individual’s age.While statistical models are by nature complex, the creation of a dashboard may easier facilitate and streamline individual assessments in routine practice. Although AI approaches are in development, providing a validated probability method addresses a knowledge gap and is of high interest as currently, no available method can provide a reliable CA.

## Supplementary Information

Below is the link to the electronic supplementary material.

Supplementary file1 (DOCX 484 KB)

Supplementary file2 (DOCX 325 KB)

Supplementary file3 (DOCX 324 KB)

Supplementary file4 (DOCX 464 KB)

Supplementary file5 (DOCX 459 KB)

Supplementary file6 (DOCX 254 KB)

Supplementary file7 (DOCX 255 KB)

Supplementary file8 (DOCX 106 KB)

Supplementary file9 (DOCX 270 KB)

Supplementary file10 (DOCX 234 KB)

Supplementary file11 (DOCX 222 KB)

Supplementary file12 (DOCX 383 KB)

Supplementary file13 (DOCX 19 KB)

Supplementary file14 (DOCX 19 KB)

Supplementary file15 (DOCX 22 KB)

Supplementary file16 (DOCX 28 KB)

## Data Availability

The source code for running all the modeling, simulations, and providing the results as well as the dashboard can be obtained by contacting the first author.
