# Correction to: A probability model for estimating age in young individuals relative to key legal thresholds: 15, 18 or 21-year

**DOI:** 10.1007/s00414-024-03347-4

**Published:** 2024-10-05

**Authors:** Nina Heldring, Ali-Reza Rezaie, André Larsson, Rebecca Gahn, Brita Zilg, Simon Camilleri, Antoine Saade, Philipp Wesp, Elias Palm, Ola Kvist

**Affiliations:** 1 Department of Forensic Medicine, Swedish National Board of Forensic Medicine, Retzius Väg 5, 171 65 Stockholm, Sweden; 2 Department of Oncology-Pathology, Karolinska Institutet, Retzius V. 3, 171 77 Stockholm, Sweden; 3 Paindrainer, Medicon Village, 223 81 Lund, Sweden; 4 Faculty of Dentistry, Oral and Craniofacial Sciences, Tower Wing, Guys’ Hospital St Thomas Street, London, England; 5 Department of Orthodontics, Faculty of Dental Medicine, Lebanese University, Beirut, Lebanon; 6 Department of Radiology, LMU University Hospital, LMU Munich, Marchioninistraße 15, 81377 Munich, Germany; 7 Munich Center for Machine Learning (MCML), Geschwister-Scholl‑Platz 1, 80539 Munich, Germany; 8 Pediatric Radiology Department, Karolinska University Hospital, Stockholm, Sweden; 9 Department of Women’s and Children’s Health, Karolinska Institute, Stockholm, Sweden

**Correction to: International Journal of Legal Medicine (2024)**

10.1007/s00414-024-03324-x

An error was identified in the original published article. On page 4 of the PDF version, in the section titled “*Data extraction and simulating population age distributions*,” the second-of-last sentence mistakenly includes the extraneous data “33333333”. The correct sentence should read: “The resulting bivariate normal distribution is then used to recreate the CA and the stages for each individual in the study”.

The original article has been corrected.

