# Supplementary material for: A probability model for estimating age in young individuals relative to key legal thresholds: 15, 18 or 21-year
Source: Int J Legal Med. 2024 Sep 18;139(1):197–217. doi: 10.1007/s00414-024-03324-x (PMC11732925; doi:10.1007/s00414-024-03324-x)
Supplement: Supplementary file 1 — Supplementary file1 (DOCX 484 KB) [file 414_2024_3324_MOESM1_ESM.docx]

**“A probability model** **for estimating age in young individuals relative to key legal thresholds: 15, 18 or 21-year.”** *International Journal of legal medicine.* Nina Heldring^1,2*^, Ali-Reza Rezaie^1^, André Larsson^3^, Rebecca Gahn^1^, Brita Zilg^1,2^, Simon Camilleri^4^, Antoine Saade^5^, Philipp Wesp^6,7^, Elias Palm^1^ and Ola Kvist^8,9^.

^1^ Department of Forensic Medicine, Swedish National Board of Forensic Medicine, Retzius väg 5, SE-171 65, Stockholm, Sweden

^2^ Department of Oncology-Pathology, Karolinska Institutet, Retzius v. 3, 171 77, Stockholm, Sweden.

^3^ Paindrainer, Medicon Village, 223 81 Lund, Sweden

^4^ Faculty of Dentistry, Oral and Craniofacial Sciences, Tower Wing, Guys’ Hospital St Thomas Street, London, England

^5^ Department of Orthodontics, Faculty of Dental Medicine, Lebanese University, Beirut, Lebanon

^6^ Department of Radiology, LMU University Hospital, LMU Munich, Marchioninistraße 15, 81377 Munich, Germany

^7^ Munich Center for Machine Learning (MCML), Geschwister‑Scholl‑Platz 1, 80539 Munich, Germany

^8^ Pediatric Radiology Department, Karolinska University Hospital, Stockholm, Sweden.

^9^ Department of Women's and Children's Health, Karolinska Institute, Stockholm, Sweden.

^*^ Corresponding author email: nina.heldring@rmv.se

**Supplementary Figure 1. KDE VS PDF**
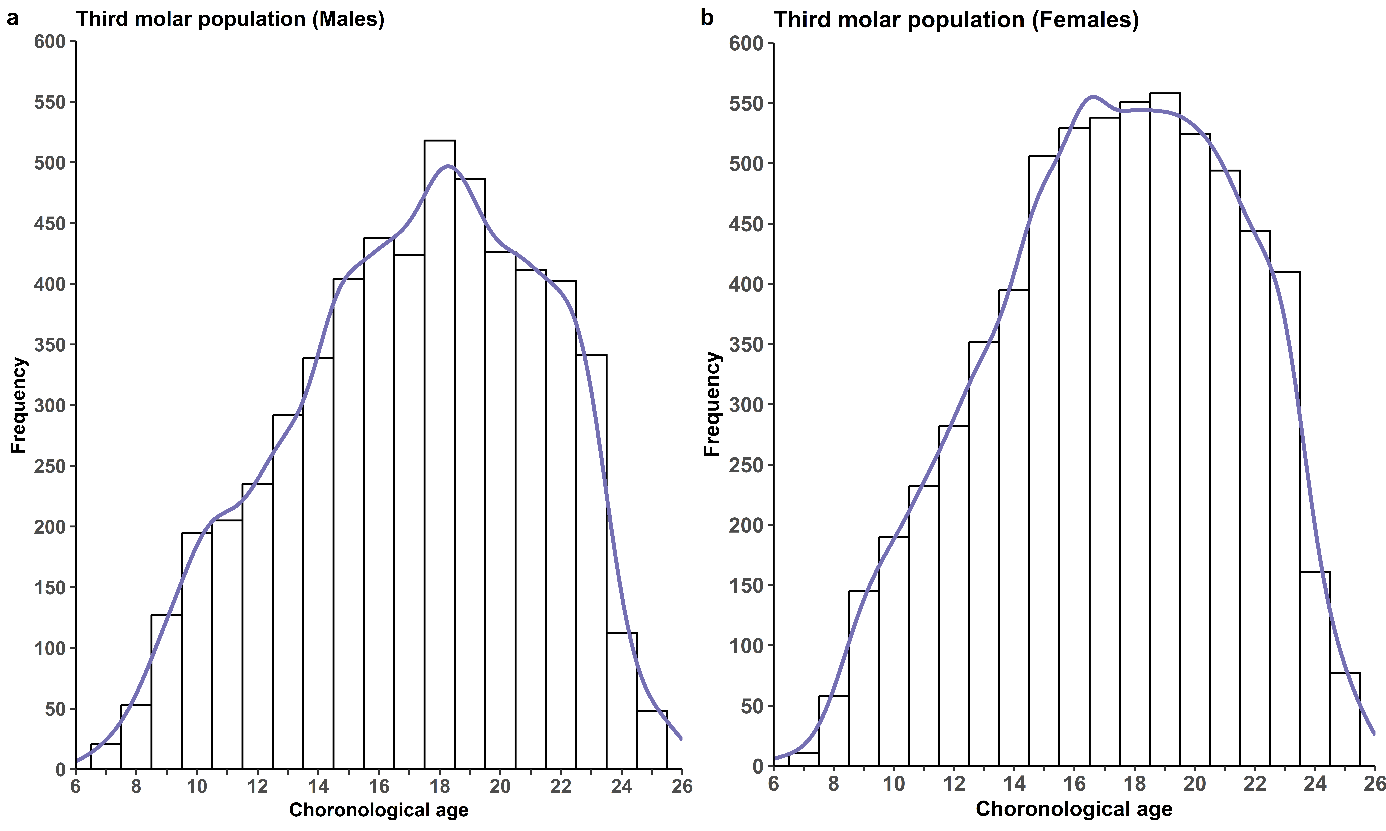


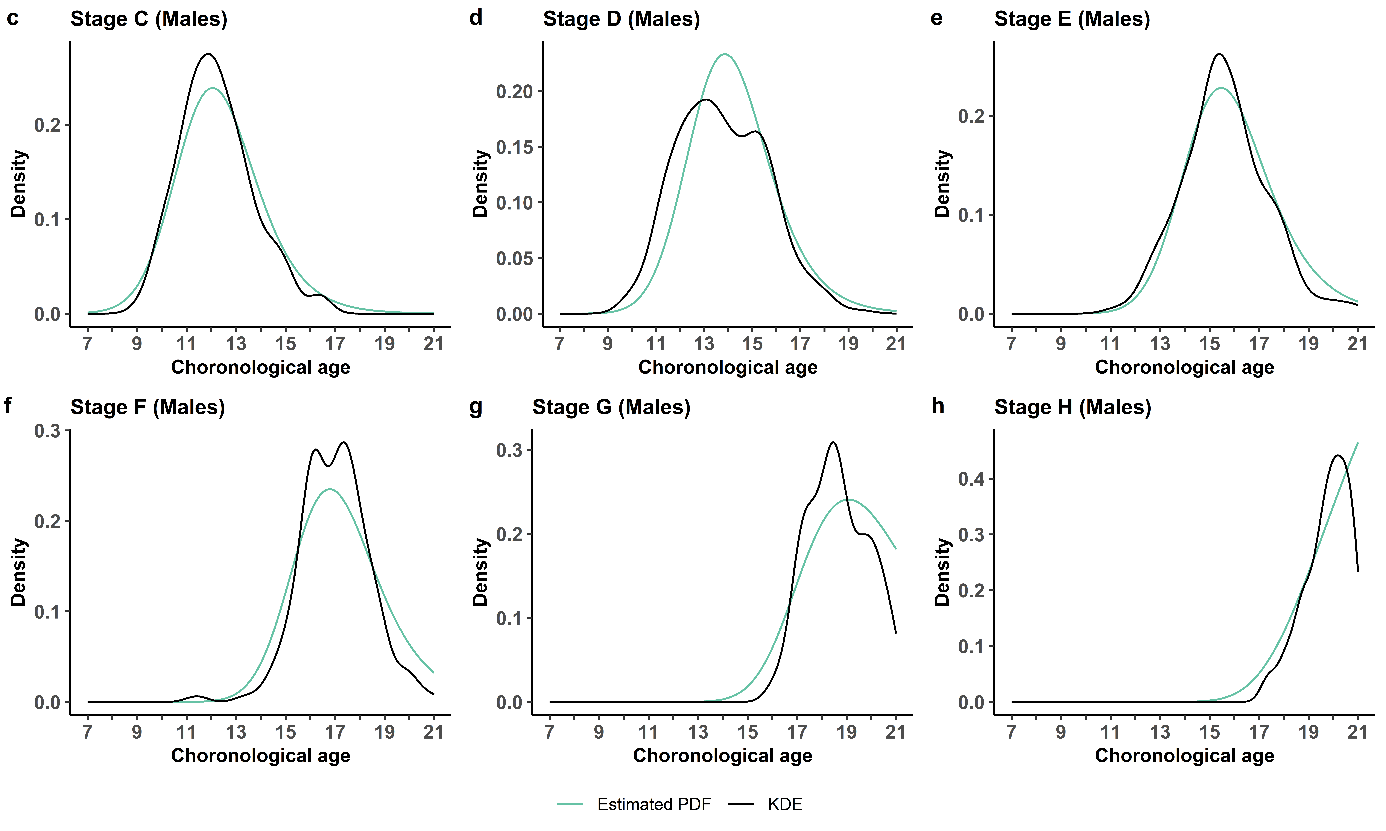

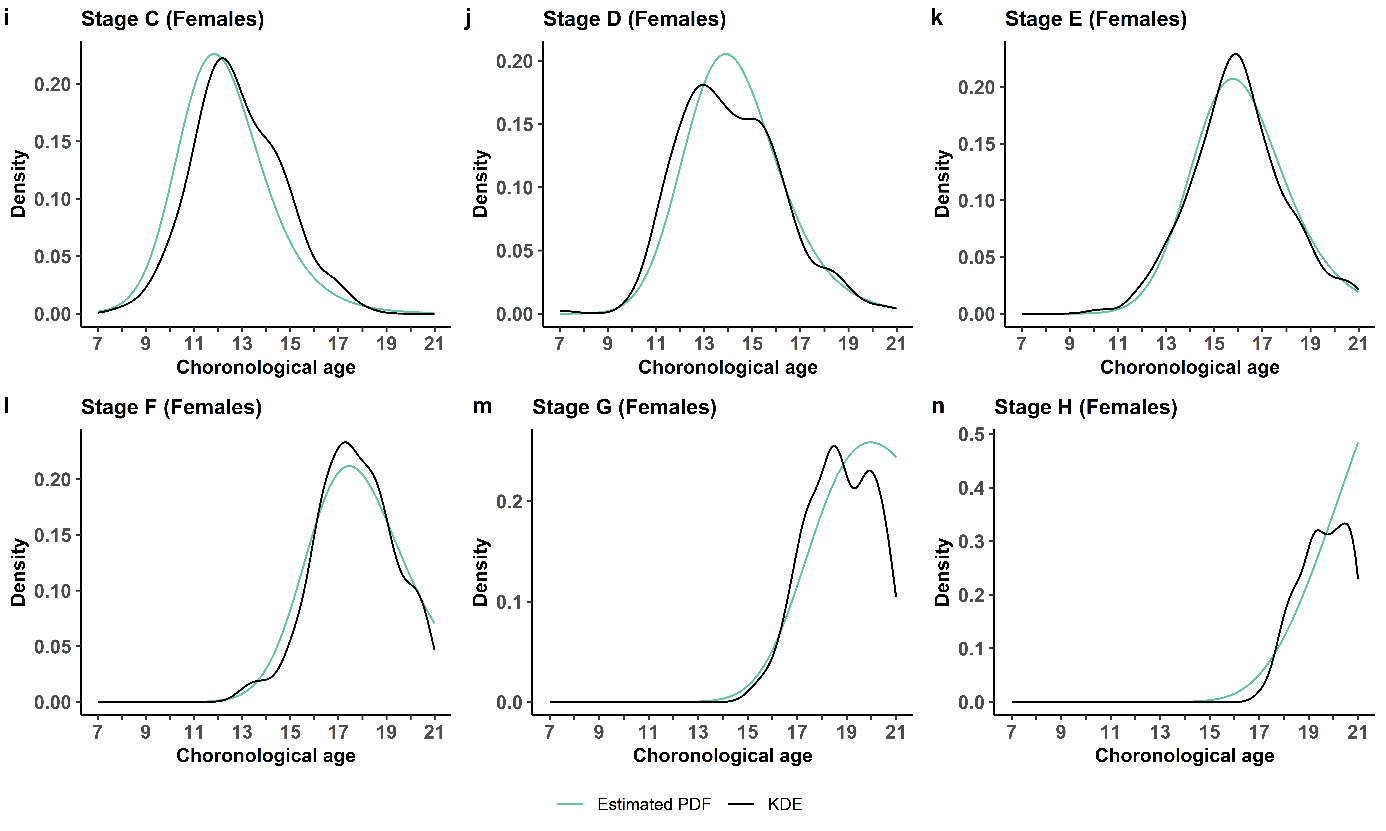
*Supplementary Figure 1. Distribution of individuals in the third molar reference population for males (a) and females (b). Kernal density estimator of the third molar validation dataset compared to the estimated conditional PDF for males (c-h) and females (i-n) illustrated with one randomly selected from 10.000 populations.*
