# Supplementary material for: A probability model for estimating age in young individuals relative to key legal thresholds: 15, 18 or 21-year
Source: Int J Legal Med. 2024 Sep 18;139(1):197–217. doi: 10.1007/s00414-024-03324-x (PMC11732925; doi:10.1007/s00414-024-03324-x)
Supplement: Supplementary file 11 — Supplementary file11 (DOCX 222 KB) [file 414_2024_3324_MOESM11_ESM.docx]

**“A probability model** **for estimating age in young individuals relative to key legal thresholds: 15, 18 or 21-year.”** *International Journal of legal medicine.* Nina Heldring^1,2*^, Ali-Reza Rezaie^1^, André Larsson^3^, Rebecca Gahn^1^, Brita Zilg^1,2^, Simon Camilleri^4^, Antoine Saade^5^, Philipp Wesp^6,7^, Elias Palm^1^ and Ola Kvist^8,9^.

^1^ Department of Forensic Medicine, Swedish National Board of Forensic Medicine, Retzius väg 5, SE-171 65, Stockholm, Sweden

^2^ Department of Oncology-Pathology, Karolinska Institutet, Retzius v. 3, 171 77, Stockholm, Sweden.

^3^ Paindrainer, Medicon Village, 223 81 Lund, Sweden

^4^ Faculty of Dentistry, Oral and Craniofacial Sciences, Tower Wing, Guys’ Hospital St Thomas Street, London, England

^5^ Department of Orthodontics, Faculty of Dental Medicine, Lebanese University, Beirut, Lebanon

^6^ Department of Radiology, LMU University Hospital, LMU Munich, Marchioninistraße 15, 81377 Munich, Germany

^7^ Munich Center for Machine Learning (MCML), Geschwister‑Scholl‑Platz 1, 80539 Munich, Germany

^8^ Pediatric Radiology Department, Karolinska University Hospital, Stockholm, Sweden.

^9^ Department of Women's and Children's Health, Karolinska Institute, Stockholm, Sweden.

^*^ Corresponding author email: nina.heldring@rmv.se

**Supplementary Figure 11. Prediction intervals of chronological age for each development stage of the clavicle and third molar models**

**
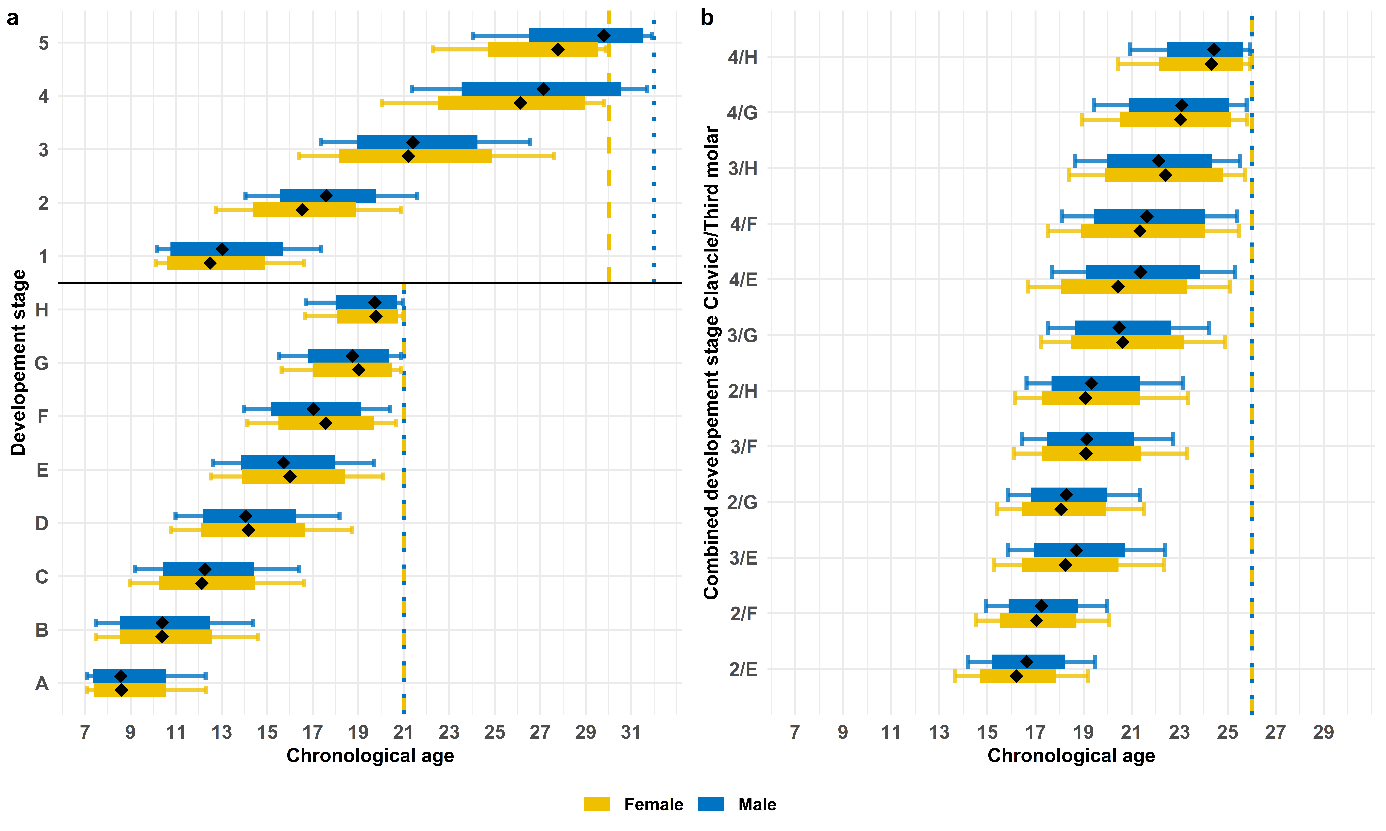
**

*Supplementary Figure 11. The figure shows the 75% (rectangles) and 95% (lines) PI of chronological age for each development stage for (a) clavicle or third molar and (b) in the combination of clavicle and third molar for males (blue) and females (yellow). The dotted vertical lines are the set upper age limits of the age prior for each indicator for males (blue) and females (yellow). The black diamonds represent the 50% percentile of the distribution for each stage or combination of stages.*
