# Supplementary material for: A probability model for estimating age in young individuals relative to key legal thresholds: 15, 18 or 21-year
Source: Int J Legal Med. 2024 Sep 18;139(1):197–217. doi: 10.1007/s00414-024-03324-x (PMC11732925; doi:10.1007/s00414-024-03324-x)
Supplement: Supplementary file 12 — Supplementary file12 (DOCX 383 KB) [file 414_2024_3324_MOESM12_ESM.docx]

**“A probability model** **for estimating age in young individuals relative to key legal thresholds: 15, 18 or 21-year.”** *International Journal of legal medicine.* Nina Heldring^1,2*^, Ali-Reza Rezaie^1^, André Larsson^3^, Rebecca Gahn^1^, Brita Zilg^1,2^, Simon Camilleri^4^, Antoine Saade^5^, Philipp Wesp^6,7^, Elias Palm^1^ and Ola Kvist^8,9^.

^1^ Department of Forensic Medicine, Swedish National Board of Forensic Medicine, Retzius väg 5, SE-171 65, Stockholm, Sweden

^2^ Department of Oncology-Pathology, Karolinska Institutet, Retzius v. 3, 171 77, Stockholm, Sweden.

^3^ Paindrainer, Medicon Village, 223 81 Lund, Sweden

^4^ Faculty of Dentistry, Oral and Craniofacial Sciences, Tower Wing, Guys’ Hospital St Thomas Street, London, England

^5^ Department of Orthodontics, Faculty of Dental Medicine, Lebanese University, Beirut, Lebanon

^6^ Department of Radiology, LMU University Hospital, LMU Munich, Marchioninistraße 15, 81377 Munich, Germany

^7^ Munich Center for Machine Learning (MCML), Geschwister‑Scholl‑Platz 1, 80539 Munich, Germany

^8^ Pediatric Radiology Department, Karolinska University Hospital, Stockholm, Sweden.

^9^ Department of Women's and Children's Health, Karolinska Institute, Stockholm, Sweden.

^*^ Corresponding author email: nina.heldring@rmv.se

**Supplementary Figure 12. Validation of the combined hand/wrist and third molar model
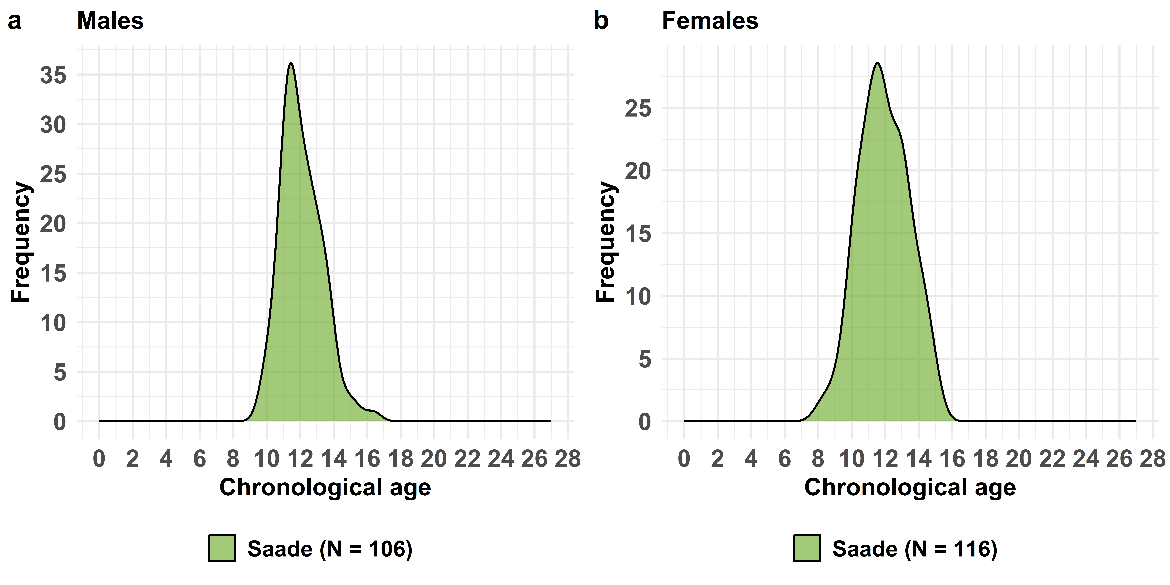
**

**
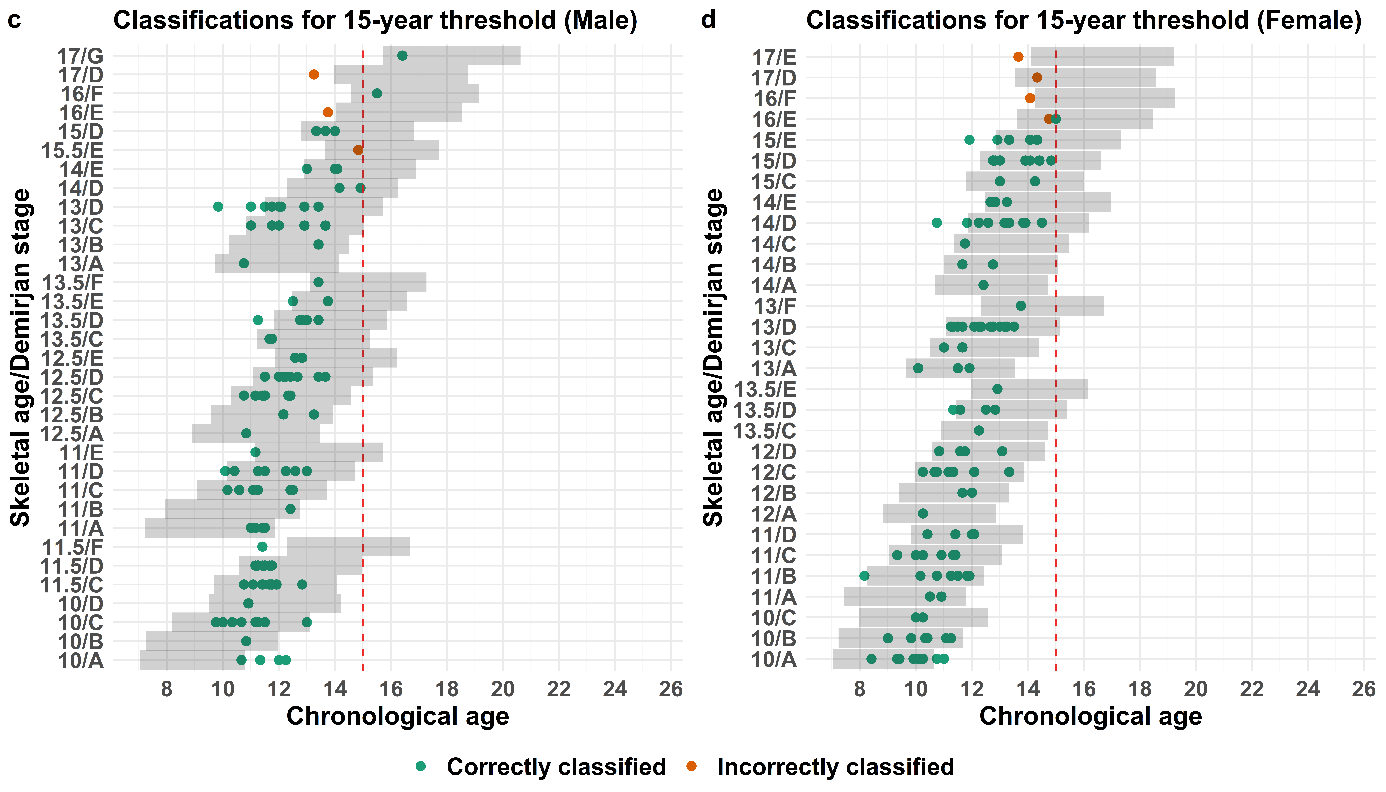
**

*Supplementary Figure 12. Validation of the combined hand/wrist and third molar model. Distribution of the full validation dataset for males(a) and females(b). Point plots displaying the chronological age and corresponding combination of hand/wrist and third molar together with classification with regard to the 15- year threshold for males (c) and females (d). Grey bars in (c and d) represents the 95% PI for each development stage in the model.*
