# Supplementary material for: A probability model for estimating age in young individuals relative to key legal thresholds: 15, 18 or 21-year
Source: Int J Legal Med. 2024 Sep 18;139(1):197–217. doi: 10.1007/s00414-024-03324-x (PMC11732925; doi:10.1007/s00414-024-03324-x)
Supplement: Supplementary file 13 — Supplementary file13 (DOCX 19 KB) [file 414_2024_3324_MOESM13_ESM.docx]

**Supplementary Table 1. Number of studies, individuals and geographic regions**

| **Indicator** | **Number of studies** | **Number of geographic regions** | **Men, number** | **Women, number** | **Total** |
| --- | --- | --- | --- | --- | --- |
| Hand/wrist | 15 | 5 | 5 431 | 4 493 | 9 924 |
| Third molar | 10 | 4 | 6 180 | 6 874 | 13 054 |
| Distal femur | 4 | 2 | 1 041 | 773 | 1 814 |
| Clavicle | 5 | 3 | 1 565 | 1 033 | 2 598 |
| Hand/wrist + Third molar | 25 | 6 | 11 611 | 11 367 | 22 978 |
| Distal femur + Third molar | 14 | 5 | 7 221 | 7 647 | 14 868 |
| Clavicle + Third molar | 15 | 6 | 7 745 | 7 907 | 15 652 |

#### *Supplementary Table 1: Number of studies, geographic regions and individuals included in the model for each indicator or combination thereof.*
