# Supplementary material for: A probability model for estimating age in young individuals relative to key legal thresholds: 15, 18 or 21-year
Source: Int J Legal Med. 2024 Sep 18;139(1):197–217. doi: 10.1007/s00414-024-03324-x (PMC11732925; doi:10.1007/s00414-024-03324-x)
Supplement: Supplementary file 15 — Supplementary file15 (DOCX 22 KB) [file 414_2024_3324_MOESM15_ESM.docx]

**“A probability model** **for estimating age in young individuals relative to key legal thresholds: 15, 18 or 21-year.”** *International Journal of legal medicine.* Nina Heldring^1,2*^, Ali-Reza Rezaie^1^, André Larsson^3^, Rebecca Gahn^1^, Brita Zilg^1,2^, Simon Camilleri^4^, Antoine Saade^5^, Philipp Wesp^6,7^, Elias Palm^1^ and Ola Kvist^8,9^.

^1^ Department of Forensic Medicine, Swedish National Board of Forensic Medicine, Retzius väg 5, SE-171 65, Stockholm, Sweden

^2^ Department of Oncology-Pathology, Karolinska Institutet, Retzius v. 3, 171 77, Stockholm, Sweden.

^3^ Paindrainer, Medicon Village, 223 81 Lund, Sweden

^4^ Faculty of Dentistry, Oral and Craniofacial Sciences, Tower Wing, Guys’ Hospital St Thomas Street, London, England

^5^ Department of Orthodontics, Faculty of Dental Medicine, Lebanese University, Beirut, Lebanon

^6^ Department of Radiology, LMU University Hospital, LMU Munich, Marchioninistraße 15, 81377 Munich, Germany

^7^ Munich Center for Machine Learning (MCML), Geschwister‑Scholl‑Platz 1, 80539 Munich, Germany

^8^ Pediatric Radiology Department, Karolinska University Hospital, Stockholm, Sweden.

^9^ Department of Women's and Children's Health, Karolinska Institute, Stockholm, Sweden.

^*^ Corresponding author email: [nina.heldring@rmv.se](mailto:nina.heldring@rmv.se)

**Supplementary Table 3. Sensitivity analysis of upper age limits for the combination of clavicle and third molar model**

| Kön | Demirjian | Clavicle stage (Schmeling) | 25 yr | 26 yr | 27 yr | 28 yr | 29 yr | 30 yr |
| --- | --- | --- | --- | --- | --- | --- | --- | --- |
| Male | F | I | 1.00 | 1.00 | 1.00 | 1.00 | 1.00 | 1.00 |
| Male | G | I | 1.00 | 1.00 | 1.00 | 1.00 | 1.00 | 1.00 |
| Male | H | I | 0.97 | 0.97 | 0.97 | 0.97 | 0.97 | 0.97 |
| Male | F | II | 0.99 | 0.99 | 0.99 | 0.99 | 0.99 | 0.99 |
| Male | G | II | 0.96 | 0.96 | 0.96 | 0.96 | 0.96 | 0.96 |
| Male | H | II | 0.84 | 0.83 | 0.83 | 0.83 | 0.83 | 0.83 |
| Male | F | III | 0.86 | 0.86 | 0.86 | 0.86 | 0.86 | 0.86 |
| Male | G | III | 0.62 | 0.61 | 0.61 | 0.61 | 0.61 | 0.61 |
| Male | H | III | 0.30 | 0.28 | 0.27 | 0.27 | 0.26 | 0.26 |
| Male | F | IV | 0.40 | 0.38 | 0.37 | 0.36 | 0.36 | 0.36 |
| Male | G | IV | 0.16 | 0.14 | 0.13 | 0.12 | 0.12 | 0.12 |
| Male | H | IV | 0.04 | 0.03 | 0.02 | 0.02 | 0.01 | 0.01 |
| Male | F | V | 0.19 | 0.16 | 0.15 | 0.14 | 0.14 | 0.13 |
| Male | G | V | 0.06 | 0.05 | 0.04 | 0.03 | 0.03 | 0.02 |
| Male | H | V | 0.01 | 0.01 | 0.00 | 0.00 | 0.00 | 0.00 |
| Female | F | I | 1.00 | 1.00 | 1.00 | 1.00 | 1.00 | 1.00 |
| Female | G | I | 0.99 | 0.99 | 0.99 | 0.99 | 0.99 | 0.99 |
| Female | H | I | 0.96 | 0.96 | 0.96 | 0.96 | 0.96 | 0.96 |
| Female | F | II | 0.99 | 0.99 | 0.99 | 0.99 | 0.99 | 0.99 |
| Female | G | II | 0.96 | 0.96 | 0.96 | 0.96 | 0.96 | 0.96 |
| Female | H | II | 0.84 | 0.84 | 0.84 | 0.84 | 0.84 | 0.84 |
| Female | F | III | 0.84 | 0.83 | 0.83 | 0.83 | 0.83 | 0.83 |
| Female | G | III | 0.58 | 0.57 | 0.56 | 0.56 | 0.56 | 0.56 |
| Female | H | III | 0.29 | 0.26 | 0.25 | 0.24 | 0.23 | 0.23 |
| Female | F | IV | 0.46 | 0.44 | 0.43 | 0.42 | 0.42 | 0.41 |
| Female | G | IV | 0.21 | 0.18 | 0.16 | 0.15 | 0.14 | 0.14 |
| Female | H | IV | 0.07 | 0.05 | 0.03 | 0.03 | 0.02 | 0.02 |
| Female | F | V | 0.27 | 0.24 | 0.22 | 0.20 | 0.19 | 0.19 |
| Female | G | V | 0.10 | 0.07 | 0.06 | 0.05 | 0.04 | 0.04 |
| Female | H | V | 0.03 | 0.02 | 0.01 | 0.01 | 0.00 | 0.00 |

#### *Supplementary Table 3: Sensitivity analysis of upper age limits. Probabilities of being under 21 when the upper age limit is varied on the uniform distribution for the combination of clavicle and third molar. The green color indicates the upper limit applied in the statistical model.*
