# Supplementary material for: A probability model for estimating age in young individuals relative to key legal thresholds: 15, 18 or 21-year
Source: Int J Legal Med. 2024 Sep 18;139(1):197–217. doi: 10.1007/s00414-024-03324-x (PMC11732925; doi:10.1007/s00414-024-03324-x)
Supplement: Supplementary file 16 — Supplementary file16 (DOCX 28 KB) [file 414_2024_3324_MOESM16_ESM.docx]

**“A probability model** **for estimating age in young individuals relative to key legal thresholds: 15, 18 or 21-year.”** *International Journal of legal medicine.* Nina Heldring^1,2*^, Ali-Reza Rezaie^1^, André Larsson^3^, Rebecca Gahn^1^, Brita Zilg^1,2^, Simon Camilleri^4^, Antoine Saade^5^, Philipp Wesp^6,7^, Elias Palm^1^ and Ola Kvist^8,9^.

^1^ Department of Forensic Medicine, Swedish National Board of Forensic Medicine, Retzius väg 5, SE-171 65, Stockholm, Sweden

^2^ Department of Oncology-Pathology, Karolinska Institutet, Retzius v. 3, 171 77, Stockholm, Sweden.

^3^ Paindrainer, Medicon Village, 223 81 Lund, Sweden

^4^ Faculty of Dentistry, Oral and Craniofacial Sciences, Tower Wing, Guys’ Hospital St Thomas Street, London, England

^5^ Department of Orthodontics, Faculty of Dental Medicine, Lebanese University, Beirut, Lebanon

^6^ Department of Radiology, LMU University Hospital, LMU Munich, Marchioninistraße 15, 81377 Munich, Germany

^7^ Munich Center for Machine Learning (MCML), Geschwister‑Scholl‑Platz 1, 80539 Munich, Germany

^8^ Pediatric Radiology Department, Karolinska University Hospital, Stockholm, Sweden.

^9^ Department of Women's and Children's Health, Karolinska Institute, Stockholm, Sweden.

^*^ Corresponding author email: nina.heldring@rmv.se

**Supplementary Appendix**

***Truncated normal***

If the random variable *X* follows a normal distribution with mean *μ* and variance *σ^2^* and it lies in the interval $\left[ a, b \right]$, where $-\infty\leq a < b \leq\infty$*,* then *X* has a truncated normal distribution conditional on $a < X < b$ [1]. The conditional PDF of the truncated normal distribution is defined as

$$f\left( x;\mu, \sigma,a,b \right)=\left\{ \begin{aligned} \frac{1}{\sigma}\frac{\varphi\left( \frac{x-\mu}{\sigma} \right)}{\Phi\left( \frac{b-\mu}{\sigma} \right)-\Phi\left( \frac{a-\mu}{\sigma} \right)}, &\mathrm{for} x\in\left[ a, b \right] \\ 0, &\mathrm{otherwise} \end{aligned} \right.$$

where $\varphi$ and $\Phi$ is the PDF and cumulative distribution function (CDF) respectively of the standard normal distribution. By definition if $a = -\infty$ then $\Phi\left( \frac{a-\mu}{\sigma} \right)=0$ and conversely if $b = \infty$ then $\Phi\left( \frac{b-\mu}{\sigma} \right)=1$. The conditional CDF of the truncated normal distribution is defined as

$$F\left( x;\mu, \sigma,a,b \right)=\left\{ \begin{aligned} 0, &x<0 \\ \frac{\Phi\left( \frac{x-\mu}{\sigma} \right)-\Phi\left( \frac{a-\mu}{\sigma} \right)}{\Phi\left( \frac{b-\mu}{\sigma} \right)-\Phi\left( \frac{a-\mu}{\sigma} \right)}, &\mathrm{for} x\in\left[ a, b \right] \\ 1, &x>b \end{aligned} \right.$$

***Type 3 data***

Each one-year age-cohorts of the type 3 study populations have to follow a uniform distribution which ensures roughly equal frequencies across ages. To verify that the requirement is fulfilled, a one-way Chi-square test (*p-value* *< 0.05*) is conducted on the age cohorts, with the alternative hypothesis that the data is not uniformly distributed. This procedure is done to limit the impact of age mimicry.

Type 3a data involves reporting mean and standard deviation for each stage *s*. For each stage, data recreation via simulations assumes a normal distribution, considering the specified age range [*a*, *b*] reported in the research article. Consequently, a truncated normal distribution is applied within this age range. However, the mean and standard deviation of the truncated normal distribution differ unless the limits of [*a*, *b*] extends to [*−∞*, *∞*], as shown above. A non-linear optimizer is utilized to derive mean *μ_t_* and standard deviation *σ_t_* for the truncated normal distribution which minimizes the distance of the reported (*μ*, *σ*) and estimated (*μ_t_*, *σ_t_*) parameters given *a* and *b*.

To be able to fit a truncated normal distribution to type 3b, a non-linear approximation was applied. The values that were used to fit the distribution was the given minimum and maximum values as [*a*, *b*], as well as the lower and upper quartile and the median, were fitted to a truncated normal. The aim is to minimize the distance between the given values and the estimated truncated normal distribution with parameters *μ_t_*, *σ_t_*, *a* and *b*. Both the 3a and 3b methods were estimated applying the *optim* function and the *truncnorm* package in R respectively.

***Candidate regression models***

The two models under consideration for data modeling are the cumulative model and the continuous-ratio (CR) model. Additionally, a critical parameter to tune when selecting a model is the choice of the link function, which can either be *logit* or *probit*. Finally, it's essential to examine the slope coefficient *β* of the regression model. This involves determining whether the model exhibits a parallel fit with the same *β* across ordered stages $j = 1, 2, ..., J - 1$, or if individual $\beta_{j}$coefficients should be estimated for each stage (except the last stage, which will be discussed further below).

In total eight different model configurations are compared. Let *α* be the intercept, *β* the slope of the model mentioned above, and *x* the chronological age. By letting *Y* be a discrete stochastic variable with ordered stages and its outcomes $j = 1, 2, ..., J$ that an individual of age *x* may be in, then the possible candidate models can be described as follows for the stages $1, 2, ..., J - 1$

1. Parallel cumulative model with *logit* link

$$logit(P\left( Y \leq j \right| \alpha_{j} , \beta, x)) =\alpha_{j} + \beta x$$

2. Parallel cumulative model with *probit* link

$$probit(P(Y \leq j | \alpha_{j} , \beta, x)) = \alpha_{j} + \beta x$$

3. Non-Parallel cumulative model with *logit* link

$$logit(P\left( Y \leq j \right| \alpha_{j} , \beta_{j} , x)) = \alpha_{j} + \beta_{j}x$$

4. Non-Parallel cumulative model with *probit* link

$$probit(P\left( Y \leq j \right| \alpha_{j} , \beta_{j} , x)) = \alpha_{j} + \beta_{j}x$$

5. Parallel CR model with *logit* link

$$logit(P(Y = j | Y \geq j, \alpha_{j} , \beta, x)) = \alpha_{j} + \beta x$$

6. Parallel CR model with *probit* link

$$probit(P(Y = j | Y \geq j, \alpha_{j} , \beta, x)) = \alpha_{j} + \beta x$$

7. Non-Parallel CR model with *logit* link

$$logit(P(Y = j | Y \geq j, \alpha_{j} , \beta_{j} , x)) = \alpha_{j} + \beta_{j}x$$

8. Non-Parallel CR model with *probit* link

$$probit(P(Y = j | Y \geq j, \alpha_{j} , \beta_{j} , x)) = \alpha_{j} + \beta_{j}x$$

For the last stage $j = J$, it is defined as $P(Y \leq j | \alpha j , \beta\backslash\beta j , x) = 1$ and $P(Y = j | Y \geq\geq j, \alpha j , \beta\backslash\beta j , x) = 1$.

For the knee indicator, the logistic regression with either a *logit* or *probit* link function is chosen. There are two stages when it comes to the logistic regression, either $P(S = 1 | A)$ which is the end stage or $P(S = 0 | A)$ which is the premature stage [2]. [3]

***Predictive intervals of ages***

The conditional PDF were derived for age given stage $P(A| S = s)$ with Bayes’ theorem. To calculate a 95% or 75% prediction interval of the age range of a indicators stage, the inverse CDF, $F^{-1}(x) = Q(x)$ was derived to extract the ages $x_{1}$ and $x_{2}$ corresponding to the 2.5%, and 97.5% or 12.5% and 87.5% percentile of the distribution. This was accomplished by employing a numeric approximation method by applying a non-linear optimizer. The implementation was carried out by utilizing the function *optim* in R along with a self-written function. First $P(A| S = s)$ was derived for a given stage, indicator (or combination) and gender, then a function $f_{min}(x)$ was created that minimized the distance between itself and the percentile of interest $Q(x)$ to find the optimal point $D(x)$ which can be stated as

$$D(x) = {(Q(x) - f_{min}(x))}^{2}$$

where $f_{min}(x)$ is defined as

$$f_{min}\left( x \right)= \int_{a}^{z} P(A| S = s) dx$$

The variable $a$ is the lower limit of the uniform prior. The non-linear optimizer finds a point for $z$ that minimizes the distance by a quasi-Newton method, this point is a close approximation of the age for that given percentile. The optimizing algorithm of choice was *L-BFGS [3]*. This was done for every 10 000 generated population and the median value was selected from the simulated distribution as the final results.

**References**

1. Nielsen F (2022) Statistical Divergences between Densities of Truncated Exponential Families with Nested Supports: Duo Bregman and Duo Jensen Divergences. Entropy (Basel) 24. doi: 10.3390/e24030421

2. Bleka O, Wisloff T, Dahlberg PS, Rolseth V, Egeland T (2019) Advancing estimation of chronological age by utilizing available evidence based on two radiographical methods. Int J Legal Med 133: 217-29. doi: 10.1007/s00414-018-1848-y

3. Byrd RH, Lu P, Nocedal J, Zhu C (1995) A Limited Memory Algorithm for Bound Constrained Optimization. SIAM Journal on Scientific Computing 16: 1190-208. doi: 10.1137/0916069
